# Molecular Characterization of c-Abl/c-Src Kinase Inhibitors Targeted against Murine Tumour Progenitor Cells that Express Stem Cell Markers

**DOI:** 10.1371/journal.pone.0014143

**Published:** 2010-11-30

**Authors:** Thomas Kruewel, Silvia Schenone, Marco Radi, Giovanni Maga, Astrid Rohrbeck, Maurizio Botta, Juergen Borlak

**Affiliations:** 1 Center for Pharmacology and Toxicology, Hannover Medical School, Hannover, Germany; 2 Department of Pharmaceutical Science, University of Genoa, Genoa, Italy; 3 Department of Chemistry and Pharmaceutical Technology, University of Siena, Siena, Italy; 4 Institute of Molecular Genetics IMG-CNR, Pavia, Italy; 5 Department of Molecular Medicine and Medical Biotechnology, Fraunhofer Institute of Toxicology and Experimental Medicine, Hannover, Germany; Hungarian Academy of Sciences, Hungary

## Abstract

**Background:**

The non-receptor tyrosine kinases c-Abl and c-Src are overexpressed in various solid human tumours. Inhibition of their hyperactivity represents a molecular rationale in the combat of cancerous diseases. Here we examined the effects of a new family of pyrazolo [3,4-d] pyrimidines on a panel of 11 different murine lung tumour progenitor cell lines, that express stem cell markers, as well as on the human lung adenocarcinoma cell line A549, the human hepatoma cell line HepG2 and the human colon cancer cell line CaCo2 to obtain insight into the mode of action of these experimental drugs.

**Methodology/Principal Findings:**

Treatment with the dual kinase inhibitors blocked c-Abl and c-Src kinase activity efficiently in the nanomolar range, induced apoptosis, reduced cell viability and caused cell cycle arrest predominantly at G0/G1 phase while western blot analysis confirmed repressed protein expression of c-Abl and c-Src as well as the interacting partners p38 mitogen activated protein kinase, heterogenous ribonucleoprotein K, cyclin dependent kinase 1 and further proteins that are crucial for tumour progression. Importantly, a significant repression of the epidermal growth factor receptor was observed while whole genome gene expression analysis evidenced regulation of many cell cycle regulated genes as well integrin and focal adhesion kinase (FAK) signalling to impact cytoskeleton dynamics, migration, invasion and metastasis.

**Conclusions/Significance:**

Our experiments and recently published *in vivo* engraftment studies with various tumour cell lines revealed the dual kinase inhibitors to be efficient in their antitumour activity.

## Introduction

Cancer research identified c-Abl and c-Src kinases to be overexpressed and to be hyperactive in various malignancies. Consequently, research is being directed towards the synthesis and characterization of novel inhibitors of these non-receptor tyrosine kinases which play important roles in various signal transduction pathways to mediate cellular growth, proliferation, invasion and metastatic spread [1], [2].

Notably, the first approved kinase inhibitor for the treatment of chronic myeloid leukaemia (CML) was imatinib (Glivec). This drug inhibits chimeric Bcr/Abl kinase, i.e. a truncated fusion protein generated by chromosomal translocation of a breakpoint cluster region (Bcr) with the Abl gene that has also been referred to as the Philadelphia chromosome in leukaemia patients. Indeed, inhibition of Bcr/Abl by imatinib prevented hyperproliferation of leukaemic cells and is considered to be a first line treatment of CML [3], [4]. However, prolonged treatment of patients resulted in therapeutic failures and chemoresistance, in part due to various mutations, such as the gate-keeper mutation that prevented the binding of imatinib to the ATP binding site [5]. Thus, a new generation of kinase inhibitors have been envisioned and research programs amongst different laboratories pursue the synthesis and evaluation of new classes of kinase inhibitors in the combat of cancer.

In this regard, the Src non-receptor tyrosine kinases (Src, Fyn, Yes, Blk, Yrk, Fgr, Hck, Lck and Lyn) received much attention and are considered to be part of the molecular basis of imatinib's resistance [6], particularly as Src kinases remain full activity after imatinib treatment [7]. To overcome imatinib's chemoresistance, dual kinase inhibitors against c-Abl and c-Src were developed and dasatinib (Sprycel) is the first generation of a new class of dual kinase inhibitors displaying striking therapeutic benefit [8], [9].

Specifically, dasatinib can be used effectively to overcome imatinib's resistance as described in detail elsewhere [10] and more than 20 clinical trials are on the way to evaluate the therapeutic benefit of either imatinib and/or dasatinib in the treatment of solid tumours [11]–[15]. Notably, inhibition of c-Src may lead to an improved chemosensitivity as was shown for patients with pancreatic cancers with resistance against 5-fluorouracil that blocks thymidylate synthase [16]. Moreover, recent advances in the treatment of hepatocellular carcinoma (HCC) with the tyrosine kinase inhibitors sorafenib (Nexavar) or sunitinib (Sutent) demonstrate the therapeutic value of multikinase inhibition [17]–[20].

Taken collectively, there is considerable evidence for c-Src and c-Abl dual kinase inhibitors to represent an important strategy in the combat of cancer. The design of novel c-Abl/c-Src inhibitors on the basis of different molecular scaffolds may improve therapeutic options in patients refractory to common protocols.

In this regard, our research group carried out extensive studies on a new family of pyrazolo [3,4-d]pyrimidines which we found to block c-Abl and c-Src phosporylation efficiently in the nanomolar range. This new class of inhibitors induce effectively apoptosis, reduce cell proliferation in different solid tumour cell lines such as epidermoid carcinoma A431 cells, the breast cancer 8701-BC cells, the osteosarcoma SaOS-2 cells and the prostate cancer PC3 cells. In addition, this new class of inhibitors were well tolerated in engraftment experiments with the epidermoid carcinoma cell lines A431, and evidence has been obtained for these compounds to be potent inhibitors of angiogenesis due to reduced production of VEGF [21]–[24].

Here we report the efficacy and the molecular pharmacology of 17 novel functionalized pyrazolopyrimidines that were studied on a panel of 11 different murine lung tumour progenitor cell lines that express stem cell markers and are derived from a cmyc/craf transgenic mouse model of lung cancer, as recently reported by us [25]. The dual kinase inhibitors were also tested in the human lung adenocarcinoma cell line A549, the human hepatoma cell line HepG2 and the human colon cancer cell line CaCo2. To improve an understanding of the mode of action of the most active inhibitors, whole genome expression analysis were carried out and subjected to advanced computational pathway analysis to enable generation of hypothesis and to validate further such data by protein expression studies.

Overall, we report the effectiveness of 17 novel dual kinase inhibitors on a large panel of epithelial tumour cell lines and provide novel insight into the mode of action of these experimental drugs.

## Results

### Cancer stem cell markers

The murine lung tumour cells A2C12, BetaD5, GammaA3 and GammaD12 were isolated from mice transgenic for *c-Myc* and *c-Raf* as described recently [26]. For comparison the human lung cancer cell line A549, the human hepatoma HepG2 and the colon carcinoma CaCo2 cells were studied as well. Initially, we investigated the expression of the cancer stem cell markers *Cd34*, *Cd24a*, *Cd44*, *Cd133*, *Cd90*, Podoplanin (*Pdpn*), Nestin (*Nes*) and Discs, large homolog 7 (Drosophila) (*Dlg7*). As shown in the supplementary table S1 expression of stem cell markers varied between the different cell lines, albeit expression was generally increased when compared to appropriate controls. These cells were used to investigate the effects of a series of dual kinase inhibitors on growth and resistance of tumours and to identify possible candidates for further preclinical development.

### c-Abl/c-Src dual kinase inhibitors

As the 17 4-amino-substituted pyrazolo[3,4-*d*]pyrimidine derivatives **4–5** and **9–23** were ATP-competitive, the dual kinase inhibitors were tested for kinase inhibition and affinity to c-Abl and c-Src. The calculated *K*i-values for c-Abl ranged in the nanomolar concentration. However, the affinity to c-Src differed considerable according to the different substitutes. While *K*i-values were predominantly obtained in a nanomolar range, some were also at the micromolar range (Table 1). Best inhibitory results were obtained for Si162 with a *K*i of 42 nM and 444 nM for c-Src and c-Abl, respectively.

**Table 1:** Allocation of substituents and their effect on enzyme kinetics of c-Abl and c-Src.

				*K*i (µM)	
Compound	R_1_	R_2_	R_3_	c-Abl	c-Src
**4** (Si 135)	SC_5_H_10_	NHC_6_H_4_mCl	CH_2_CHClC_6_H_5_	0.120	NA
**5** (Si 142)	SCH(CH_3_)_2_	NHCH_2_CH_2_C_6_H_5_	CH_2_CHClC_6_H_5_	0.120	2.000
**9** (Si 166)	CH_3_	NHC_6_H_4_mF	CH_2_CHClC_6_H_5_	0.151	0.338
**10** (Si 167)	CH_3_	NHC_6_H_4_mCl	CH_2_CHClC_6_H_5_	0.500	0.362
**11** (Si 20)	SC_2_H_5_	NHC_4_H_9_	CH_2_CHClC_6_H_5_	0.320	0.600
**12** (Si 27)	SCH_3_	N(C_2_H_5_)_2_	CH_2_CHClC_6_H_5_	0.440	0.500
**13** (Si 34)	SCH_3_	NHCH_2_C_6_H_5_	CH_2_CHClC_6_H_5_	0.260	3.700
**14** (Si 81)	SCH_3_	NHCH_2_C_6_H_5_	CH_2_CHBrC_6_H_5_	0.190	1.800
**15** (Si 65)	SCH_3_	NHCH_2_C_6_H_5_	CH_2_CHClC_6_H_4_pF	0.250	35.000
**16** (Si 66)	SCH_3_	NHCH_2_CH_2_C_6_H_5_	CH_2_CHClC_6_H_4_pF	0.200	5.300
**17** (Si 93)	SCH_3_	NHC_6_H_4_mF	CH_2_CHClC_6_H_4_pF	0.220	4.510
**18** (Si 40)	SCH_3_	NHCH_2_CH_2_C_6_H_5_	CHCHC_6_H_5_	0.450	NA
**19** (Si 57)	SCH_3_	NHCH_2_CH_2_C_6_H_4_pCl	CH_2_CHClC_6_H_5_	0.350	25.000
**20** (Si 70)	SCH_3_	NHCH_2_C_6_H_5_	CH_2_CHClC_6_H_4_pCl	0.110	3.000
**21** (Si 51)	H	4-morpholino	CH_2_CHBrC_6_H_5_	0.220	NA
**22** (Si 104)	H	NHC_6_H_4_mCl	CH_2_CHClC_6_H_4_pF	0.180	1.400
**23** (Si 162)	H	NHCH_2_C_6_H_4_oF	CH_2_CHClC_6_H_5_pBr	0.444	0.042

*K*i values toward isolated Abl and Src were calculated according to the following equation: 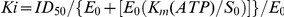 where *E*_0_ and *S*_0_ are the enzyme and the ATP concentrations (0.005 and 0.012 µM. respectively).

For all inhibitors the cytotoxicity was determined by use of the MTS-assay, that is a colorimetric assay of cell viability and based on the reduction of a tetrazolium salt (MTS) by a mitochondrial reductase, at concentrations of 1, 10 and 100 µM after single treatment for 24 h (data not shown). Based on this initial screening, the murine tumour-derived cell lines (A2C12, BetaD5, GammaA3 and GammaD12) and the human tumour cell lines (A541, CaCo2 and HepG2) were selected for in depth investigations.

An IC_50_ for each of the dual kinase inhibitors, as well as for the approved kinase inhibitors imatinib mesylate and dasatinib, was determined after treatment for 24 or 96 h. Clear evidence was obtained for structurally related compounds to differ in their cytotoxic potential. (Note, the IC_50_ after single treatment for 24 h are given in supplementary table S2).

Except for the human hepatoma HepG2 tumour cell line, the IC_50_ for lung tumour cells and the human colon carcinoma cell line CaCo2 were in the range of 3 to 12 µM.

Amongst the individual dual kinase inhibitors Si135 and Si162 were most effective. In the case of Si162 and depending on the tumour cell line studied the IC_50_ ranged between 0.8 and 6.4 µM. However, with the HepG2 cell line an IC_50_ of 14.5 µM was calculated (see supplementary table S3 for IC_50_ values after 96 h).

### Cell cycle analysis

After monitoring the cytotoxic potential of the dual kinase inhibitors, the effects on cell cycle regulation were analyzed by flow cytometry at IC_50_ treatment conditions in response to daily treatment for 96 h.

Notably, those Si-compounds with high potency such as Si162 also induced most significant changes in the cell cycle. Compared to the vehicle treatment that consisted of DMSO (0.2%) only a decrease in the S-phase of up to 91% and an increase in G0/G1 of up to 92% (GammaA3 treated with Si70) was determined. A similar change was reported for dasatinib after treatment of various tumour cell lines. Note, this is an approved c-Src and c-Abl inhibitor [27]. With Si162 an increase in the G2/M phase (up to 72.3%±2.8% of all A549 cells) was determined in lung tumour and hepatoma cancer cell lines, respectively (see supplementary table S4).

### Caspase activity

**Figure 1:**
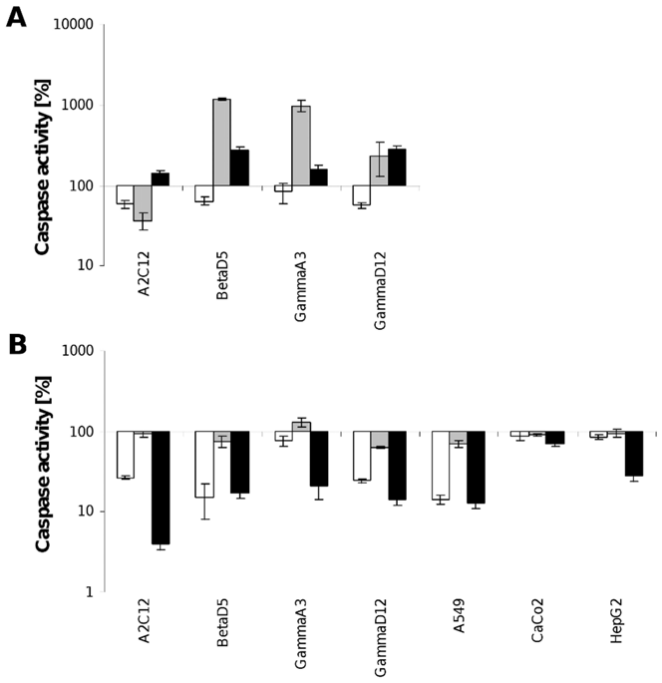
Caspase-3/7-activity. Activity of caspases 3 and 7 is shown in reference to vehicle after treatment with the dual kinase inhibitors Si57 (white bars), Si135 (grey bars) and Si162 (black bars) for 24 h (A) and 96 h (B). Caspase activity of vehicle cells was defined as 100%.

Accompanied by significant changes in cell cycle regulation caspase-3/7-activity increased strongly. Caspase activity was evaluated with the most active inhibitors (Si57, Si135 and Si162). Clear differences between these experimental dual kinase inhibitors and the induction of caspase activity was observed. This difference in response is depicted in Fig. 1.A. Strikingly, after treatment with Si57 the caspase activity declined in all cell lines, while treatment with Si135 caused a 10 fold increase in caspase activity as determined for the BetaD5 and GammaA3 cell lines. With Si162 caspase activity increased in all tested cell lines up to 3 fold after treatment as determined for GammaD12. After 96 h of treatment (Fig. 1.B) caspase activity returned to normal or was below control values, except for Si135 and the cell line GammaA3 where an increase of about 30% of control was recorded.

Together, these results indicate the high cytotoxic potential of the tested dual kinase inhibitors. Treatment with the inhibitors led predominantly to cell cycle arrest in G0/G1, however Si162 caused an arrest in G2/M. This suggests inference of kinase inhibitors at different phases of the cell cycle, that coincided with induction of caspase activity.

### Western blot analysis

**Figure 2:**
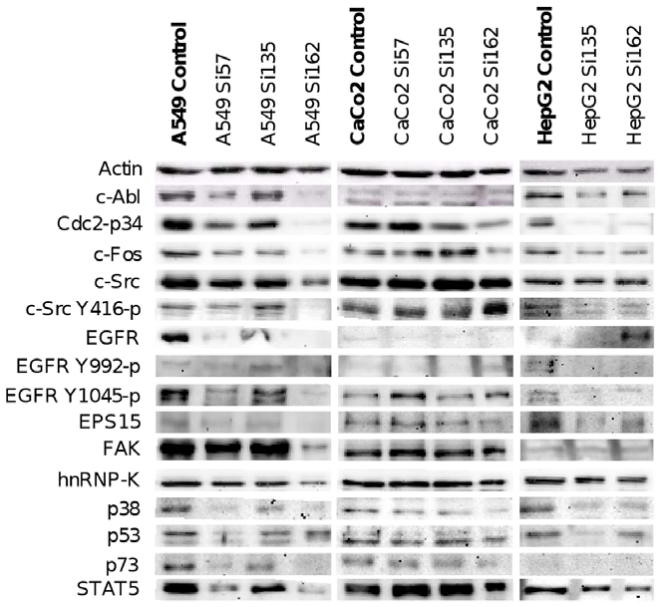
Treatment effects on the protein concentration in human cell lines. Cells were seeded in various densities in 75 cm^2^ cell culture flasks. After 72 h adhesion, the cells were treated with the dual kinase inhibitors for 96 h. Culture media enriched with experimental compounds (IC_50_ concentrations) was exchanged daily. After harvest, preparation of SDS extract and SDS-PAGE with an amount of 25 µg protein, Western blot analyses were carried out.

**Figure 3:**
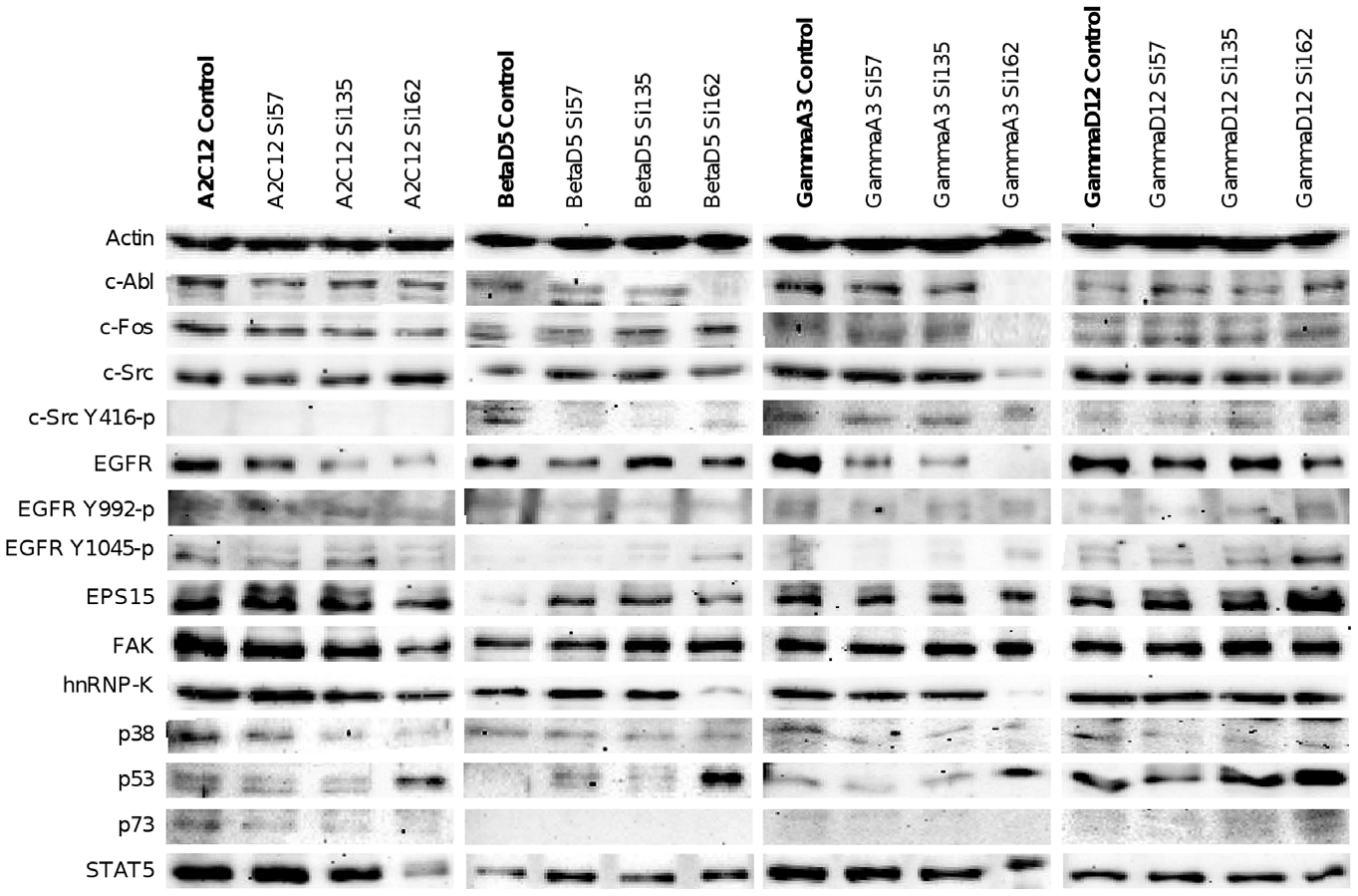
Treatment effects on the protein concentration in murine cell lines. Cells were seeded in various densities in 75 cm^2^ cell culture flasks. After 72 h adhesion, the cells were treated with the dual kinase inhibitors for 96 h. Culture media enriched with experimental compounds (IC_50_ concentrations) was exchanged daily. After harvest, preparation of SDS extract and SDS-PAGE with an amount of 25 µg protein, Western blot analyses were carried out.

Western blots were carried out with all human (Fig. 2) and murine (Fig. 3) cell lines after treatments with Si57, Si135 and Si162 respectively, at IC_50_ concentrations determined for the 96 h time point. Compared to vehicle treated controls, the dual kinase inhibitors repressed protein expression of c-Abl and c-Src up to 90%. Nonetheless, the posttranslational modification of c-Src was basically unchanged and the level of activated and phosphorylated c-Src on residue Tyr416 remained equal. However, the lung tumour cell lines A549 and BetaD5 displayed inhibition of c-Src autophosphorylation after treatment with Si162. A further important finding of the Western blot experiments was the repression of EGFR, an upstream molecule in the signalling pathway of c-Src and c-Abl. Indeed, its expression was remarkably reduced after treatment with the dual kinase inhibitors. However, no changes in the phosphorylation of EGFR residue Tyr992 were detectable. Contrary to Tyr992, the phosphorylation of residue Tyr1045 indicates heterogeneity towards cell line and treatment; likewise, the epidermal growth factor receptor substrate 15 (EPS15) displayed heterogeneity of response to treatment.

An other Src substrate, the focal adhesion kinase (FAK), was clearly repressed (up to 50%) after treatment with Si162 in lung cancer cell lines. Equally, the downstream p38 MAPK was 80% less expressed after treatment. The detection of proteins Cdc2-p34, c-Fos, hnRNP-K, p53, p73 and STAT5 gave further insight on the condition of the cells after inhibition of c-Abl and c-Src. The antibody against Cdc2, better known as cyclin dependent kinase 1 or p34, was reduced up to 95% after treatment. Note, this kinase plays pivotal roles in G1/S and G2/M transitions [28], [29] and activates c-Src, by phosphorylation of serine and threonine residues, when cells enter mitosis [30]. Most distinct reduction of Cdc2 was detected after treatment with Si162, and this finding agrees well with the observed G2/M arrest of treated cell lines. Again, Si162 was more potent than Si135 but the level of heterogenous nuclear ribonucleoprotein K (hnRNP-K), that plays a role in facilitating c-Src phosphorylation, remained equal after treatment with Si57 and Si135. Note, c-Src is a substrate of hnRNP-K [31] and the phosphorylation by c-Src drives the translational activation of hnRNP-K [32].

The protein expression of the transcription factors c-Fos and STAT5 was also reduced by approximately 90% and 80%, respectively for A549. Both are c-Src mediated downstream targets of EGFR and crucial for tumour progression [33], [34]. A clear induction of p53 could be observed after treatment with individual dual kinase inhibitors albeit at different level when different cell lines and treatment conditions were compared. The antibody targeted against p73 generated moderately detected after treatment of A549 and CaCo2 tumour cells. The absence of cleaved PARP product (data not shown) agreed well with the results obtained for caspase activity which declined after multiple treatment for 96 h, therefore suggesting that induction of apoptosis is an initial and timed event.

### Whole genome expression analysis

**Figure 4:**
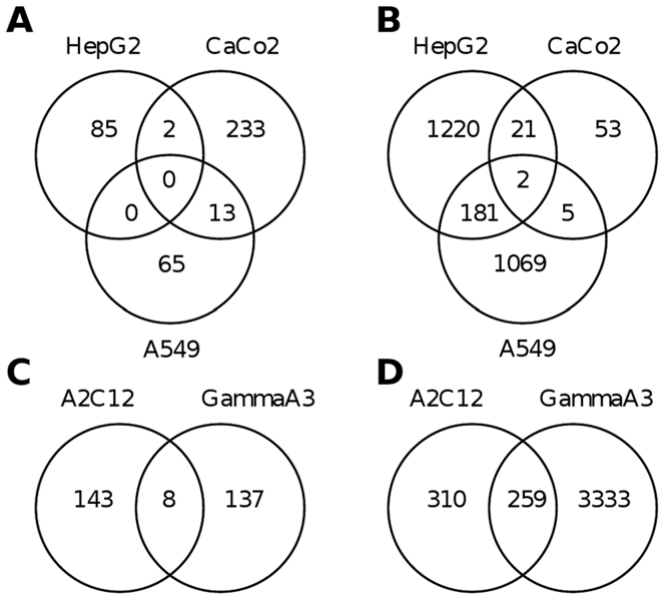
Commonly regulated genes after treatments with the dual kinase inhibitors. Commonly regulated genes in human cell lines after treatment with Si135 (A) and Si162 (B), and in murine cell lines after treatment with Si135 (C)and Si162 (D) were displayed as venn-diagrams.

The two most sensitive murine (A2C12 and GammaA3) and the three human cell lines were treated with the most active dual kinase inhibitors Si135 and Si162 at IC_50_ concentrations for 96 h. Then, microarray experiments were performed and analyzed using the ArrayTrack software (National Center for Toxicological Research/FDA, Jefferson, AR, USA). Essentially, the data was analyzed by two-class unpaired Significance Analysis of Microarrays (SAM). Genes with a False Discovery Rate (FDR)<0.01, a Mean Channel Intensity (MCI)>100, Bad Flags less or equal 2 and a Fold Change (FC)>2 were considered as significant. Highly significant changes in gene expression were determined (Table 2), however neither treatment with Si135 nor Si162 revealed any alterations in gene expression of the two target kinases c-Abl and c-Src. After treatment with Si135 approx. 150 genes were significantly regulated, whereas with Si162 more than 3500 and 500 genes were regulated in the cell lines GammaA3 and A2C12, respectively while only 75 genes were regulated in CaCo2 cells. Both murine cell lines shared a relatively large intersection of 259 commonly regulated genes (Fig. 4D), while the human cell lines responded to this treatment differently. After treatment with Si135, there was no common gene regulated in any of the human cancer cell lines tested. Note, the cancer cell lines A549 and CaCo2 shared regulation of 13 genes in common (Fig. 4A). That is approx. 16% of all differentially regulated genes in A549 but only about 5% of those in CaCo2. After treatment with the dual kinase inhibitor Si162 there were only two genes regulated in common amongst all three human cell lines, namely Serpine peptidase inhibitor, clade E, member 2 (*Serpine2*) and tubulin, alpha 1a (*Tuba1a*) (Fig. 4B). The significantly regulated genes were classified according to their biological functions thereby revealing a significant change in expression of genes involved in the process of cell communication and cell cycle regulation. To enable hypothesis generation and to better understand the biological significance of differentially expressed genes, specific regulatory and signalling pathway networks were constructed with the Ingenuity Pathway Analysis tool (Ingenuity Systems, Mountain View, CA, USA; www.ingenuity.com).

**Table 2:** Significantly differentially regulated genes after treatment with Si135 and Si162.

	Si135		Si162	
	downregulated	upregulated	downregulated	upregulated
A2C12	33	118	212	357
GammaA3	66	79	1139	2453
A549	17	61	472	785
CaCo2	55	193	6	75
HepG2	0	87	707	717

After treatment with Si135 and Si162 at IC_50_ concentrations for 96 h, whole genome expression analyses were carried out. Microarray data was analyzed with ArrayTrack software. Significantly differentially regulated genes were determined by two-class unpaired SAM with an FDR<0.01; MCI>100; Bad Flags: 2; FC>2.

Indeed, microarray data revealed in lung tumour and hepatoma cell lines distinct regulations in integrin and FAK (focal adhesion kinase) signalling, as well as altered actin dynamics (see supplementary tables S5 and S6). As c-Src and c-ABL play pivotal roles in cytoskeleton rearrangements, various cellular functions such as migration, metastasis, invasion and mitosis may be affected. Specifically FAK is crucial for centrosome function during mitosis [35] and the increased expression of *Gadd45a* and several kinase inhibitors such as *p21Cip1*, *p15Ink4*, *p16Ink4* and *p19Arf*, as well as the repression of Cdc2 (Cdk1), may explain, at least in part, the effects of dual kinase inhibitors on spindle formation in mitosis. Furthermore, the gene expression analysis revealed an upregulation of programmed cell death inducing genes coding for calpain, p53 apoptosis effector related to PMP-22 (*PERP*), Caspase 6, p53-induced protein with a death domain (*PIDD*), PMA-induced protein 1 (*NOXA*) and Bcl2-like protein 11 (*Bim*). In the case of p53, a strong protein induction was confirmed as well (Fig. 2 and 3) as was activity of caspase 3/7 by flow cytometry.

The treatment effects of Si135 were less pronounced as observed with Si162, therefore demonstrating the importance of the molecular structure in causing different biological effects. After the treatment with the dual kinase inhibitor the cells predominantly arrested in G0/G1 phase as determined for GammaA3 where up to 75% of cells remained in this phase. All treated cell lines displayed a change in expression pattern of genes coding for proteins of the cytoskeleton and proteins involved in cell growth and migration which we found to be repressed. Additionally, treatment with Si135 altered the expression of cell cycle regulators and inhibited the signal transduction via mitogen activated protein kinases (MAPK) that was also evidenced for p38 at the protein level (Fig. 2 and 3). Together, the results suggest the cell cycle arrest to be in part by induction of kinase inhibitors like *p21Cip1* and *Gadd45a* and such cell cycle arrest coincided with elevated caspase activity as part of a programmed cell death.

### Secondary effects with the dual kinase inhibitors

The networks around the tyrosine kinases c-Src and c-Abl as well as EGFR and HGF/c-Met were constructed and analyzed.

#### Cell line A549 treated with Si162 (see supplementary figure S1)

Treatment of A549 lung cancer cells with Si162 caused induction of a large number of genes, but only a few were downregulated. It is of considerable importance that transcript expression of the kinases *c-Abl* and *c-Src* were unchanged, while expression of genes coding for DNA-damage response and checkpoint regulation were downregulated. Indeed, regulation of *Rad51*, essential for homologous recombination as well as breast cancer 1 (*Brca1*) and Fanconi anemia, complementation group A (*Fanca*) that build DNA repair complexes were found to be repressed. Further genes associated with DNA repair that were repressed were DNA-directed polymerase, delta 1, catalytic subunit (*Pold1*), origin recognition complex, subunit 1-like (*Orc1l*) and topoisomerase (DNA) II binding protein 1 (*Topbp1*). Additionally, the cell cycle regulators cell division cycle 2 (*Cdc2*), phosphatase cell division cycle 25c (*Cdc25c*), cyclin-dependent kinase inhibitor 3 (*Cdkn3*) and polo-like kinase 1 (*Plk1*) were downregulated. Note, the latter kinase is frequently overexpressed in tumour cells and represents a molecular target in cancer therapy. The apoptosis inhibitor baculoviral IAP repeat-containing 5 (*Birc5*), also known as survivin, which is highly expressed in lung tumours was significantly repressed upon treatment with dual kinase inhibitors while members of the Wnt-pathway such as glycogen synthase 3 beta (*Gsk3b*) or diacylglycerol kinase alpha (*Dgka*), Inositol-polyphosphat-5-phosphatase (*Inpp5d*) and prostaglandin-endoperoxide synthase 2 (*Ptgs2*) were upregulated, as was expression of *Jun*, early growth response (*Egr1*), *Elf3* and *Ehf3*.

Conversely, genes that are widely overexpressed in tumours like Mucin 1 (*Muc1*) [36], the protease cathepsin B (*Ctsb*) [37] and integrin, beta 4 (*Itgb4*) remained upregulated upon treatment with the dual kinase inhibitor. Molecules that are linked to cell-cell contact like E-cadherin (*Cdh1*) and vitronectin (*Vtn*) were also induced as was the induction of the p53 inhibitor *Mdm2*, that binds to p53 and prevents its activation as part of a negative feedback autophosphorylation [38]. This demonstrates that Si162 regulates only certain cancer genes in the A549 tumour cell line within the c-Src and c-Abl network.

#### Cell line A2C12 treated with Si162 (see supplementary figure S2)

Treatment of this murine lung cancer cell line with Si162 did not alter gene expression of the target kinases Abl, EGFR, Met and Src while an increased protein expression of tumour suppressor *p53* is consistent with the toxic effects caused by Si162. Downregulation of cyclin A2 (*Ccna2*), Polo-like kinase 1 (*Plk1*) and the centromer protein A (*Cenpa*) which are typically upregulated in tumour cells to foster cell cycle and mitosis agree well with the observed cell cycle arrest and demonstrate the therapeutic effect of these experimental inhibitors. Indeed, downregulation of ERBB feedback inhibitor receptor 1 (*Errfi1*), whose expression is elevated in cell growth, provides further evidence for this dual kinase inhibitor to cause cell cycle arrest. Several growth factors were downregulated as well like osteoglycin (*Ogn*), pleiotrophin (*Ptn*) and transforming growth factor, beta 3 (*Tgfb3*) that in turn regulate transcription factors like serum response factor (*Srf*), transforming growth factor beta 1 induced transcript 1 (*Tgfb1i1*) and nuclear factor I/B (*Nfib*). The functional relationship between Src inhibition and regulation of the receptor tyrosine kinase platelet-derived growth factor receptor beta (*Pdgfrb*) [39] as well as the fibronectin receptor integrin alpha 5 (*Itga5*) [40] has been commonly observed in tumour cells. In the network of c-Abl and c-Src and similar to the observations described for the human lung cancer cell line A549, an induced expression of *Mdm2* and *Gadd45a* was noted, as was an induction of the matrix metallopeptidases 3 and 13 (*Mmp3* and *Mmp13*) that are involved in metastasis to support degradation of extracellular matrix proteins [41]. Furthermore, treatment with Si162 altered expression of genes involved in Wnt and Toll-like pathways. Thus, expression of the receptors toll-like receptor 4 (*Tlr4*) and secreted frizzled-related protein 1 (*Sfrp1*) were upregulated and might be linked to an induced expression of the cytokines secreted phosphoprotein 1 (*Spp1*) and chemokine (C-C motif) ligand 5 (*Ccl5*). Importantly, expression of chemokine (C-X-C motif) ligand 12 (*Cxcl12*) which plays an essential role in tumour migration remained downregulated [42]–[44].

#### Cell line GammaA3 treated with Si162 (see supplementary figure S3)

Treatment with the dual kinase inhibitor Si162 resulted in more than 3500 differentially expressed genes and about 100 molecules in the context of the tyrosine kinases c-Abl, EGFR, c-Met and c-Src. Additionally, genes involved in DNA checkpoints (e.g. Brca1) and repair (e.g. Rad51) were significantly regulated, such as those involved in the G2/M checkpoint.

#### Cell line CaCo2 treated with Si162 (see supplementary figure S4)

In contrast to the lung cancer cell lines, the human colon adenocarcinoma cell line CaCo2 differed in its response to treatment with Si162. Especially metabolic pathways e.g. fatty acid metabolism was regulated. The network around the tyrosine kinases shows clear differences to the lung cancer cell lines. Notably, only induced genes were found in the network around the target kinases, but included upregulated *Ceacam6* and metastasis-involved molecules such as *Mmp1* and *Cd44* [45], [46]. Additionally, genes coding for the cytoskeleton such as tubulin alpha 1a (*Tuba1a*) and vimentin (*Vim*), a member of the intermediate filament family, as well as cytokines *Spp1* and *Ccl20* were increased after treatment with Si162. Notably, expression of caveolin 1 (*Cav1*) and AXL receptor tyrosine kinase (*Axl*) is of therapeutic importance as is *Cav1*, known as tumour suppressor where it functions as a negative regulator of the Ras-p42/44 MAP kinase cascade. On the other side, Cav1 also supports the initial activation in the Ras-ERK signalling by mediating the binding of integrin subunits on the FYN tyrosine kinase [47], [48].

#### Cell line HepG2 treated with Si162 (see supplementary figure S5)

Treatment of the human hepatocellular carcinoma cell line HepG2 with Si162 resulted in regulation of p53 and DNA repair, most notably the G2/M checkpoint. Overall, more than 1400 genes were differentially expressed. Although expression of *Src* and *Abl* were unchanged at the mRNA level several downstream kinases were regulated and this included downregulated epidermal growth factor receptor family *Erbb4* but induction of *Egfr*. Further downregulated receptor tyrosine kinases were platelet-derived growth factor receptor beta (*Pdgfrb*) and insulin receptor (*Insr*). Expression of the serine/threonine kinase *Akt2* was also repressed as was the gene expression of phosphorylase kinase alpha 2 (*Phka2*), an enzyme in carbohydrate metabolism, and phosphoglycerate kinase that is involved in glycolysis. Notably, treatment of HepG2 with Si162 induced expression of *Fas*, Caspase 3 (*Casp3*) and Bcl 2-like 1 (*Bcl2l1*), that act as inhibitor or activator of apoptosis depending on the posttranslational modification. Likewise myeloid cell leukaemia sequence 1 (*Mcl1*) a member of the proapoptotic Bcl-family was induced. As observed with the lung cancer cell line, the cell cycle regulator *Cdc2*, *Fanca* and *Cdc25c* were downregulated, while the kinase inhibitors *Cdkn1a* and *Cdkn2a* as well as cyclin-dependent kinase 5 (*Cdk5*) were upregulated as was expression of the p53-inhibitor *Mdm2*.

#### Cell line A549 treated with Si135 (see supplementary figure S6)

The effects with this dual kinase inhibitor were less pronounced. The network around the target kinases involves seven molecules. Notably, expression of the tumour marker *Ceacam6* and *Cdcp1* were upregulated as was prostaglandin synthase 2 (*Ptgs2*) that might lead to a modified arachidonic acid metabolism pertinent to the respiratory epithelium [49]. Furthermore, altered expression of the cell adhesion molecule laminin beta 3 (*Lamb3*), the matrix-associated binding protein discs, large (Drosophila) homolog-associated protein 4 (*Dlgap4*) and versican (*Vcan*) signified the effects of this dual kinase inhibitor on cell adhesion.

#### Cell line A2C12 treated with Si135 (see supplementary figure S7)

Considering the network around the target kinases, the dual specificity phosphatase 1 (*Dusp1*) comes into focus that represses signalling through the MAPK kinases. Further cell growth regulating phosphatases are myotubularin 1 (*Mtm1*) and protein tyrosine phosphatase receptor type G (*Ptprg*) that were upregulated. Despite the considerable differences in the treatment effects of Si162 and Si135 there are also commonalities. The Wnt receptor *Sfrp1* is induced after treatment with both substances as was *Gadd45a* and matrix metallopeptidase *Mmp13*.

#### Cell line GammaA3 treated with Si135 (see supplementary figure S8)

The effects of Si135 on the cell line GammaA3 are similar to those described for A2C12 and A549. An induced gene expression of matrix metallopeptidases *Mmp3* and *Mmp13*, the centromer protein A (*Cenpa*) and integrin alpha 6 (*Itga6*) suggests interference with extracellular matrix and integrin receptors. Notably, neural guanine exchange factor (*Ngef*), that directly interacts with *Src*, was induced at the transcript level. In addition, genes involved in cell growth and proliferation such as the mitogen-activated protein kinase 8 interacting protein 1 (*Mapk8ip1*) which prevents MAPK8-mediated activation of transcription factors and interacts directly with *Src* and *Egfr* was repressed. Tyrosine-3-monooxygenase activating protein (*Ywhaz*) as well as transforming growth factor beta regulator 4 (*Tbrg4*) and Catenin delta 1 (*Ctnnd1*) which also participate in signal transduction were repressed as was regulation of collagen, type I, alpha 1 (*Col1a1*) and hyaluronan synthase 2 (*Has2*) which are crucial for cell migration and cell growth.

#### Cell line CaCo2 treated with Si135 (see supplementary figure S9)

As observed with other cancer cell lines the tumour markers *Ceacam6* as well as *Ceacam1* were upregulated in cell line CaCo2. The latter is described as mediator in cell adhesion in processes like angiogenesis, apoptosis and metastasis [50]. The induction of the tumour markers fits with the repression of metastasis suppressor 1 (*Mtss1*). In contrast, thrombospondin 1 (*Thbs1*), that is associated with tumour growth and angiogenesis, was downregulated and there was modulation of the cytoskeleton as evidenced by altered gene expression of laminin gamma 2 (*Lamc2*), filamin C gamma (*Flnc*), tubulin alpha 1a (*Tuba1a*) and actin gamma 2 (*Actg2*). Treatment of this cell line with Si135 induced expression of *Cdkn1a*, *Cav1* and *Dusp1*, that control cell cycle and progression, while *Cdc25c* and the survival factor *Birc5* were downregulated. The induction of DNA-damage inducible transcript 3 (*Ddit3*), a transcription factor for expression of DNA repair genes, might be an adaptive response to programmed cell death in tumour cells.

#### Cell line HepG2 treated with Si135 (see supplementary figure S10)

When compared with lung cancer cell lines, the human hepatoma cell line HepG2 differed in its response to treatment with Si135 and included upregulated insulin-like growth factor (*Igf*) and integrin-linked kinase (*Ilk*) that are reported to be induced in hepatocellular carcinoma [51]. The network around the target molecules c-Src and c-Abl contains only proteins with increased gene expression. Interestingly, the heteronuclear ribonucleoprotein R (*Hnrnpr*), involved in many different functions including transport of pre-mRNA from the nucleus to cytoplasm, was induced by Si135 treatment. An induction of protein tyrosine kinase 2 (*Ptk2*) and phosphoinositol-3-kinase class 2 alpha (*Pik3c2a*) was also observed. The latter is a cytoplasmic tyrosine kinase belonging to the Src-family kinases and is activated by Src through phosphorylation [52]. *Pik3c2a* is involved in various signal cascades like proliferation and migration. Additionally induced genes were cell cycle controlling proteins *Dusp1* and neural precursor cell expressed, developmentally down regulated 9 (*Nedd9*) and Tensin 1 (*Tns1*) that is involved in cytoskeleton organization.

## Discussion

The development of effective and targeted therapies for the treatment of cancer remains the challenge to society and is frequently confounded by chemoresistance resulting in low rates of remission. There is a need for improved treatment strategies and dual kinase inhibitors targeting tumour cells may provide the means to combat cancer. Here we report the effects of a family of dual c-Src/c-Abl tyrosine kinase inhibitors with a pyrazolo[3,4-d]-pyrimidine scaffold which we found to be active against different solid tumour cell lines that also express various cancer stem cell markers.

Notably, by performing genome-wide expression analysis, the molecular activities beyond dual kinase inhibitors could be hypothesised. All compounds clearly inhibited activity of the kinases c-Abl and c-Src in the nanomolar range, while for 15 out of the 17 compounds tested, IC_50_ values for cell viability were in the low micromolar range. Apart from the changes in viability and morphology, shifts in the cell cycle were observed, which is consistent with an arrest in G0/G1-phase or at G2/M transitions. A highly induced caspase 3 and 7 activity was verified after 24 h, but the efficiency of individual compounds differed amongst single cancer cell lines. Upon treatment with the dual kinase inhibitors a remarkable repression of EGFR was observed and this tyrosine kinase in considered to be upstream of the c-Src kinase. Importantly, inhibition of EGFR would be of major therapeutic advantage, such as in patients with lung cancer where EGFR is frequently hyperactive, as will be discussed below.

### c-Src/c-Abl dual kinase inhibitors induce cell cycle arrest

The investigated experimental drugs led to an cell cycle arrest in the G0/G1-phase, except for Si162 that caused an G2/M-arrest while flow cytometry analysis allowed no firm conclusions on the checkpoint regulation, the microarray and Western blot data clearly evidenced the G2/M checkpoint to be activated if DNA damage occurred in late S- or G2-phase. Thus, cell cycle is halted to allow the repair of damaged DNA. The activation of the checkpoint is decisively dependent on the inhibition of Cdc2. This kinase is activated by phosphorylation on residue Thr161 of Cdk-activating kinase (CAK) and by dephosphorylation of phosphatase Cdc25C on residues Tyr15 and Thr14. Note, Cdc25C is phosphorylated on residue Ser216 by checkpoint kinases Chk1 and Chk2, therefore representing a binding site for 14-3-3βεζ. The complex of Cdc25C and 14-3-3 is exported from the nucleus into the cytoplasm. Consequently, Cdc2 remains inactive and the cells arrest in G2 [53]. Furthermore, p53 is stabilized by phosphorylation and activates transcription of Gadd45 and p21Cip that prevent the activation of Cdc2/cyclinB. Additionally, p53 acts as transcriptional repressor of *Cdc2* and *Cyclinb* [54]. The G2/M checkpoint activation fits the experimental observations. At the protein level a clear reduction of Cdc2 could be demonstrated (Fig. 2). The reduced gene expression of *Cdc2* and *Cdc25b/c* as well as the high protein level of p53 and the induced expression of *Gadd45a* and *p21Cip1* provides clear evidence for the G2/M checkpoint activation.

The arrest in G0/G1 phase and induction of apoptosis caused by all other experimental dual kinase inhibitors, has also been observed with the dual kinase inhibitors dasatinib [55], [56] or ZD6474 [57].

### Influences of c-Src inhibition on up and downstream interacting partners

Src-family kinases are signal transducer, that are activated by various classes of cell surface receptors. They interact with a variety of molecules and mediate various cellular processes, thus mainly cell growth, proliferation and cytoskeletal rearrangements incorporating various signalling cascades like PDGFR, EGFR, FGFR, Integrins and FAK [58]. An inhibition of c-Src could be observed after treatment with Si162 in lung cancer cell lines. The level of phosphorylated c-Src (Tyr416) decreased. Residue Tyr416 is phosphorylated (and activated) by the autophosphorylation domain of c-Src. Remarkable was the reduction of c-Src protein, but the gene expression of *Src* was unaffected. Additionally, a reduction of EGFR protein was evidenced. Notably, undue activation of the EGFR may result from a combination of activating mutations in the kinase domain and by overexpression of the receptor and its ligands [59]. In addition to EGFR, c-Src is also overexpressed in NSCLC. c-Src binds the activated intracellular domain of EGFR and thus is temporarily activated. c-Src activates EGFR by phosphorylation on residue Tyr845 [60] that is located in the activation loop of the catalytic domain. The phosphorylation is essential for complete catalytic and biological activity [61]. The inhibition of c-Src showed an opposite effect on EGFR and its downstream molecules STAT3 or STAT5. A decreased protein expression of STAT5 could be verified in Western blot analysis. Considering the results obtained from microarray studies, it becomes obvious that *Src* and *Egfr* were not altered. This is highly remarkable because a reduction in protein concentration usually causes an induction in gene expression. Possibly, a feedback mechanism is interrupted. In regards to the human hepatocellular carcinoma cell line HepG2 an induction of *Egfr* gene expression was observed, that verifies the elevated protein expression.

After treatment with Si135 as well as Si162 various genes affecting cytoskeletal dynamics were altered in their expression. c-Abl and c-Src activity are crucial for growth factor and integrin signalling that induces reorganization of the cytoskeleton. Important substrates are amongst others the Rho family, GTPases and FAKs. The latter indicated also a decreased protein expression after treatment with Si162 (Fig. 2 and 3. These reactions are important for cell growth, migration and proliferation [62]. Taken collectively, the tested dual kinase inhibitors efficiently inhibited cell growth and proliferation due to the inhibition of the kinases c-Abl and c-Src.

### Effect of the tested compounds on NSCLC, HCC and colorectal carcinoma

The cell lines displayed considerable heterogeneity in response to treatment with dual kinase inhibitors with Si162 yielding best results with the human and murine lung tumour and hepatoma cell lines, whereas the response to Si135 was equally in all cell lines tested.

The IC_50_ values of the dual kinase inhibitors Si162 and Si135 were close to each other but their effects differed amongst the cancer cell lines tested. For Si162, the inhibition of c-Abl and c-Src caused an arrest at G2/M prior presumably due to repression of Cdc2 (Cdk1), activation of p53 and induction of *Gadd45a* and *p21Cip1*. In addition, the inhibition of c-Abl and c-Src caused various effects to the cytoskeleton, leading to impaired spindle formation.

Induced by DNA damage, c-Abl activates stress-activated protein kinases (SAPK), as well as janus kinase and p38 MAPK. Furthermore, an activation of p73 by phosphorylation through p38 MAPK has been reported to foster an induction of apoptosis [63]. As evidenced by Western blot, p73 was unchanged while p38 MAPK was decreased but p53 was strongly induced to suggest a strong apoptotic signal, possibly due to its ability to interfere with cytoskeleton dynamics.

### Comparison of approved and experimental dual kinase inhibitors

Since the discovery of the pathogenic *Bcr-Abl* translocation in chronic myeloid leukaemia (CML) the number of rationally designed drugs increased constantly. Imatinib (Glivec, STI571; Novartis) was the first selective tyrosine kinase inhibitor approved for the treatment of CML [64]. It is reported to inhibit the chimeric Bcr/Abl kinase with an IC_50_ of 527 nM [65], whereas the antiproliferative effect for leukaemia cells was in the submicromolar range [66]. For comparison, imatinib's IC_50_ was determined between 10 and 30 µM for all investigated cell lines after 24 h and 96 h of treatment. After repeated treatment of tumour cell lines no decline of the IC_50_ was marked. Note, an IC_50_ of 2.7 and 5.0 µM was calculated for the dual kinase inhibitors Si135 and Si162 after 96 h of treatment. Furthermore, imatinib is a highly selective ATP-competitive inhibitor of Bcr/Abl, c-KIT and platelet-derived growth factor receptor (PDGFR) [67], but the IC_50_ ranges considerably amongst different tumour cell lines, since imatinib interacts with non-conserved amino acid residues neighbouring the ATP-binding site [68]. Imatinib treatment causes the inhibition of the autophosphorylation of Bcr/Abl, c-KIT and PDGFR, that in turn leads to antiproliferative effects and induction of apoptotic activity [69]. The inhibition of Bcr/Abl prevents an antiapoptotic pathway trough STAT5-mediated Bcl-X_L_ expression [70]. The downregulation of Bcl-X_L_ is reported to release cytochrome-*c* from the mitochondria, resulting in the activation of caspase 3 [71].

Imatinib is approved for first-line treatment in CML [72], but patients become resistant to this treatment due to point mutations in the *Abl* gene resulting in substitutions of amino acid residues in the Abl protein sequence. Additionally, mutations in various domains of the Abl kinase were described [73]. Strategies to overcome the resistance are imatinib dose-escalation or adjustment based on pharmacokinetic assessment, that in turn can lead to resistance mechanisms like the amplification of *Bcr-Abl* gene or increased expression of ATP-binding cassette transporters (ABC) for drug efflux [73].

Patients that fail on imatinib therapy are treated with dasatinib (Sprycel, BMS354825), a small-molecule ATP-competitive inhibitor [74]. In contrast to imatinib, dasatinib is a dual specific c-Abl and c-Src inhibitor and prevents activation of Bcr/Abl and Src family kinases at an calculated IC_50_ of 1.83 nM. Furthermore, dasatinib selectively inhibits the kinases PDGFR, c-KIT and EPHB4. The antiproliferative effect of dasatinib to NSCLC cell lines is highly variable with IC_50_ values ranging from 80 nM to 10,000 nM [75]. This agrees well with the findings of the present study and dasatinib after treatment for 96 h. Note, Si162 displays similar activity to that of dasatinib. Especially for the murine progenitor cell line GammaD12 and the human lung cancer cell line A549 identical IC_50_ values with both agents at 800 nM and 5µM, respectively could be determined. Conversely, with the murine tumour cells GammaA3, dasatinib has a 20-fold lower IC50 than Si162.

Notably, cells treated with dasatinib show a cell cycle arrest in G0/G1 phase and undergo apoptosis [76]. The inhibition of Src-family kinases leads to changes in cell morphology due to missing activation of the Src-interacting partners FAK, paxillin, p130Cas as well as STAT3 and STAT5. Furthermore, dasatinib inhibits the activation of EGFR that in turn prevents the activation of ERK and AKT pathways [77], which strongly affects proliferation and cell survival.

Dasatinib has a broad spectrum of antiproliferative effects to overcome imatinib resistance. In contrast to imatinib it has less rigid conformational requirements to Bcr/Abl, so it inhibits the active and inactive conformation and overcomes most mutations of Bcr/Abl. Additionally, dasatinib is not a substrate of ABC-transporters [78].

Dasatinib is approved for the treatment of imatinib-resistant CML and Philadelphia (Ph+) adult lymphoblastic leukaemia (ALL) and may proof to be beneficial in solid tumours in regards to migration and invasion [79]. Even though dasatinib exhibits high potency, resistance to dasatinib was reported as well. A mutation in amino acid residue 315 (Thr to Ile) can not be overcome by dasatinib, as well as others [80].

Here we investigated dual kinase inhibitors which are structurally related to the broadly used pyrazolo[3,4-d]pyrimidines (PP1 and PP2) Src-family inhibitors. Indeed, PP2 inhibitors display similar effects as observed after treatment of tumour cell lines with the experimental drugs Si135 and Si162, including inhibition of c-Src, followed by a reduced activation of cortactin and paxillin [81]. The structures of PP2, Si135 and Si162 differ in the formation of their substitutes, however, it is not trivial to predict the conformation of the experimental drug in the ATP-binding site of the kinase.

Compared to the approved leukemic anti-cancer agents imatinib and dasatinib, our experimental dual kinase inhibitors suggests a therapeutic advantage on various types of cancer. Importantly, more than 20 clinical trials are ongoing to evaluate the therapeutic efficiency of the dual kinase inhibitors imatinib and dasatinib on various solid tumours under various therapeutic regimes and treatment conditions, e.g. a combined administration of dasatinib and ketoconazole to patients suffering on various solid tumours [82] or the treatment of advanced NSCLC with dasatinib and erlotinib [83].

As discussed above, the herein presented dual kinase inhibitors modulate cytoskeleton dynamics to cause a change in morphology. They also display strong inhibition of the EGFR protein, which is a novel finding. Affecting EGFR is of great benefit in the combat of cancer and possibly inhibiting kinases that phosphorylate EGFR may provide the means to control, at least in part, expression of the EGFR.

The characteristics of cell death between Si135 and Si162 might in part be dependent on the different inhibitory effect on the kinases c-Abl and c-Src. For Si135 a low *K*i for c-Abl, but no inhibition of c-Src was observed, while Si162 inhibits both kinases, especially c-Src at a very low *K*i value (Table 1). Further evidence stems from the gene expression data with c-Src kinase affecting the organization of the cytoskeleton as mediated by FAK, Akt and ERK amongst others. However, the predominantly c-Abl inhibitor Si135 induced cytoskeletal modifications as well, but much fewer genes were regulated and less distinct effects on cell cycle regulations were observed.

Together, the inhibition of the tyrosine kinases c-Abl and c-Src suggests a high potential for treatment of solid cancers. The dual kinase inhibitors were found active against a large panel of tumour cell lines including human and murine lung, hepatoma and colon cancer cell lines. Treatment with these experimental drugs led to growth arrest and induction of apoptosis.

## Methods

### Chemistry

Starting materials were purchased from Aldrich-Italia (Milan, Italy).

Melting points were determined with a Büchi 530 apparatus and are uncorrected. IR spectra were measured in KBr with a Perkin-Elmer 398 spectrophotometer. ^1^H NMR spectra were recorded in a (CD_3_)_2_SO solution on a Varian Gemini 200 (200 MHz) instrument. Chemical shifts are reported as δ (ppm) relative to TMS as internal standard, *J* in Hz. ^1^H patterns are described using the following abbreviations: s = singlet, d = doublet, t = triplet, q = quartet, quint = quintex, sx = sextet, m = multiplet, br = broad. All compounds were tested for purity by TLC (Merck, Silica gel 60 F_254_, CHCl_3_ as the eluant). Analyses for C, H, N, S were within ±0.3% of the theoretical value.

### Synthesis of the dual kinase inhibitors

**Figure 5:**
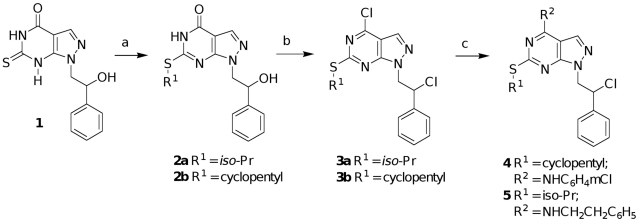
Synthesis of compounds 4 and 5. Reagents and conditions: a) RBr, K_2_CO_3_, DMF, r.t, 8h; b) POCl_3_/DMF, CHCl_3_, reflux, 8h; c) Method A: 3-chloroaniline, ethanol, reflux, 4h (for **4**); Method B: phenylethylamine, toluene, r.t.,48h (for **5**).

The synthesis of compounds **11–23** has already been reported [84]–[87], while the synthesis of compounds **4** and **5** (Table 1) is depicted in figure 5. The 1-(2-hydroxy-2-phenylethyl)-6-thioxo-1,5,6,7-tetrahydro-4*H*-pyrazolo[3,4-*d*]pyrimidin-4-one **1**, prepared according to this procedure [88], was alkylated on the C6 sulphur atom using the appropriate alkyl bromide and anhydrous K_2_CO_3_ in anhydrous dimethylformamide (DMF) at room temperature. The 6-alkylthio derivatives **2a–b** were in turn chlorinated with the Vilsmeier complex (POCl_3_∶DMF, 1∶1, 10 equiv), in CHCl_3_ at reflux for 8 h to obtain the dichloro analogues **3a–b** in good yield after chromatography purification on a Florisil column. The desired pyrazolo[3,4-d]pyrimidine **4** was obtained by reaction between **3a** and 3-chloroaniline in ethanol at reflux for 4 h (Method A) while the analogue **5** was obtained by reaction of **3b** with phenylethylamine in anhydrous toluene at rt for 2 days (Method B).

**Figure 6:**
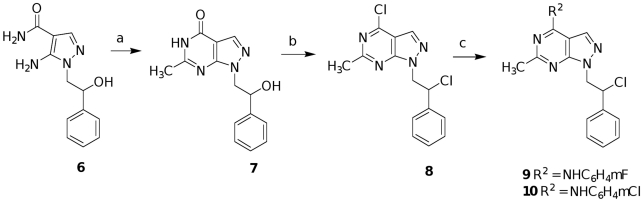
Synthesis of compounds 9 and 10. Reagents and conditions: a) EtONa, CH_3_COOEt, ethanol, reflux, 6h; b) POCl_3_/DMF, CHCl_3_, reflux, 12h; c) m-fluoro aniline (for **9**) or m-chloro aniline (for **10**), ethanol, reflux, 4 h.

Compounds **9** and **10** were prepared as described in figure 6. The 5-amino-1-(2-hydroxy-2-phenylethyl)-1*H*-pyrazole-4-carboxamide **6**, prepared according to our procedure [89], was treated with sodium ethoxide and ethyl acetate in absolute ethanol at reflux for 6 h to afford the 1-(2-hydroxy-2-phenylethyl)-6-methyl-1,5-dihydro-4*H*-pyrazolo[3,4-*d*]pyrimidin-4-one **7**, that was in turn chlorinated with the Vilsmeier complex (POCl_3_∶DMF, 1∶1, 30 equiv), in refluxing CHCl_3_ at reflux for 12 h to obtain the dichloro derivative **8**. Reaction of the latter with the suitable aniline in absolute ethanol at reflux for 4 h gave the desired compounds **9** and **10** (method A).

#### General procedure for the synthesis of 6-(alkylthio)-1-(2-hydroxy-2-phenylethyl)-1,5-dihydro-4H-pyrazolo[3,4-d]pyrimidin-4-ones (2a–b)

A mixture of 1-(2-hydroxy-2-phenylethyl)-6-thioxo-1,5,6,7-tetrahydro-4*H*-pyrazolo[3,4-*d*]pyrimidin-4-one **1** (2.88 g, 10 mmol), the appropriate alkyl bromide (10.14 mmol) and anhydrous K_2_CO_3_ (1.38 g, 10 mmol) in anhydrous DMF (10 ml) was stirred at room temperature for 8 h. The mixture was poured in cold water; the white solid was filtered, washed with water and recrystallized from absolute ethanol to afford **2a–b** as white solids.

#### 2a

Yield 37%, mp 194–195°C. ^1^H NMR: δ 1.44 (d, *J* = 6.2, 6H, 2CH_3_), 3.90 (d, 1H, OH, disappears with D_2_O), 3.98 (sept, *J* = 6.2, 1H, CHS), 4.38–4.48 and 4.50–4.58 (2m, 2H, CH_2_N), 5.19–5.22 (m, 1H, CHOH), 7.24–7.37 (m, 5H Ar), 8.06 (s, 1H, H-3), 11.06 (br s, 1H, NH disappears with D_2_O). IR (cm^−1^): 3300–3100 (NH+OH), 1704 (CO). Anal. (C_16_H_18_N_4_O_2_S) C, H, N, S.

#### 2b

Yield 55%, mp 213–214°C. ^1^H NMR: δ 1.42–1.74 and 1.94–2.27 (2m, 8H, 4CH_2_ cyclopentyl), 3.76–3.97 (m, 1H, CHS), 4.17–4.42 (m, 2H, CH_2_N), 4.92–5.12 (m, 1H, CHOH), 5.57 (d, 1H, OH disappears with D_2_O), 7.03–7.28 (m, 5H Ar), 7.87 (s, 1H, H-3), 12.19 (br s, 1H, NH disappears with D_2_O). IR (cm^−1^): 3150–2850 (NH+OH), 1703 (CO). Anal.(C_18_H_20_N_4_O_2_S) C, H, N, S.

#### General procedure for the synthesis of 6-(alkylthio)-4-chloro-1-(2-chloro-2-phenylethyl)-1H-pyrazolo[3,4-d]pyrimidines (3a–b)

The Vilsmeier complex, previously prepared from POCl_3_ (1.53 g, 10 mmol) and anhydrous DMF (0.73 g, 10 mmol) was added to a suspension of the appropriate compound **2a–b** (1 mmol) in CHCl_3_ (50 ml). The mixture was refluxed for 8 h. The solution was washed with iced water (2×20 ml), dried (MgSO_4_), filtered, and concentrated under reduced pressure. The crude oil was purified by column chromatography (Florisil, 100–200 mesh) using diethyl ether as the eluant, to afford the pure products **3a–b** as white solids.

#### 3a

Yield 74%, mp 67–68°C. ^1^H NMR: δ 1.45 (d, *J* = 6.2, 3H, CH_3_), 1.49 (d, *J* = 6.2, 3H, CH_3_), 4.00 (sept, *J* = 6.2, 1H, CHS), 4.74–4.82 and 4.88–4.97 (2m, 2H, CH_2_N), 5.45–5.55 (m, 1H, CHCl), 7.21–7.50 (m, 5H Ar), 8.00 (s, 1H, H-3). Anal. (C_16_H_16_N_4_Cl_2_S) C, H, N, S.

#### 3b

Yield 63%, mp 70–71°C. ^1^H NMR: δ 1.54–1.85 and 2.10–2.32 (2m, 8H, 4CH_2_ cyclopentyl), 3.93–4.07 (m, 1H, CHS), 4.67–4.80 and 4.82–4.97 (2m, 2H, CH_2_N), 5.38–5.50 (m, 1H, CHCl), 7.19–7.42 (m, 5H Ar), 7.94 (s, 1H, H-3). Anal. (C_18_H_18_N_4_Cl_2_S) C, H, N, S.

#### Synthesis of 1-(2-hydroxy-2-phenylethyl)-6-methyl-1,5-dihydro-4H-pyrazolo[3,4-d]pyrimidin-4-one (7)

To a solution of 5-amino-1-(2-hydroxy-2-phenylethyl)-1*H*-pyrazole-4-carboxamide **6** (2.46 g, 10 mmol) in absolute ethanol (90 mL), a solution of sodium ethoxide prepared from sodium (1.84 g, 80 mmol) and absolute ethanol (30 mL) and ethyl acetate (7.93 g, 90 mmol) were added. The mixture was refluxed for 6 h; after cooling, ice water was added and the solution was acidified with 3% acetic acid. The precipitated solid was filtered, washed with water and recrystallized from absolute ethanol to afford compound **7** as a white solid, yield 60%, mp 253–254°C. ^1^H NMR: δ 2.25 (s, 3H, CH_3_), 4.07–4.20 and 4.27–4.42 (2m, 2H, CH_2_N), 4.92–5.06 (m, 1H, CHOH), 5.52 (br s, 1H, OH disappears with D_2_O), 7.10–7.30 (m, 5H Ar), 7.90 (s, 1H, H-3), 11.91 (br s, 1H, NH disappears with D_2_O). IR (cm^−1^): 3400–3150 (NH+OH), 1660 (CO). Anal. (C_14_H_14_N_4_O_2_) C, H, N.

#### Synthesis of 4-chloro-(2-chloro-2-phenylethyl)-6-methyl-1H-pyrazolo[3,4-d]pyrimidine (8)

The Vilsmeier complex, previously prepared from POCl_3_ (4.60 g, 30 mmol) and anhydrous DMF (2.20 g, 30 mmol) was added to a suspension of 1-(2-hydroxy-2-phenylethyl)-6-methyl-1,5-dihydro-4*H*-pyrazolo[3,4-*d*]pyrimidin-4-one **7** (0.27 g, 1 mmol) in CHCl_3_ (10 ml). The mixture was refluxed for 12 h. The solution was washed with water (2×20 ml), dried (MgSO_4_), filtered and concentrated under reduced pressure. The crude oil was purified by column chromatography (Florisil, 100–200 mesh), using diethyl ether as the eluant, to afford **8** as a yellow oil, which crystallized standing in a refrigerator by adding a 1∶1 mixture of diethyl ether/petroleum ether (bp 40–60°C) as a white solid, yield 67%, mp 96–97°C. ^1^H NMR: δ 2.69 (s, 3H, CH_3_), 4.68–4.81 and 4.90–5.04 (2m, 2H, CH_2_N), 5.39–5.51 (m, 1H, CHCl), 7.16–7.41 (m, 5H Ar), 8.01 (s, 1H, H-3). Anal. (C_14_H_12_N_4_Cl_2_) C, H, N.

### General procedure for the synthesis of compounds 4, 5, 9, 10

#### Method A (4, 9, 10)

To a solution of the appropriate 4-chloro derivative **3b** and **8** (10 mmol) in absolute ethanol (10 ml) the appropriate aniline (20 mmol) was added and the mixture was refluxed for 4 h. After cooling, the white solid was filtered, washed with water and recrystallized from absolute ethanol.

#### 4

Yield 54%, mp 237–238°C. ^1^H NMR: δ 1.41–1.70 and 2.02–2.23 (2m, 8H, 4CH_2_ cyclopentyl), 3.87–4.03 (m, 1H, SCH), 4.62–4.73 (m, 2H, CH_2_N), 5.51–5.64 (m, 1H, CHCl), 7.03–7.10, 7.20–7.44, 7.52–7.63 and 8.00–8.09 (4m, 9H Ar), 8.23 (s, 1H, H-3). IR (cm^−1^): 2937 (NH). Anal. (C_24_H_23_N_5_Cl_2_S) C, H, N, S.

#### 9

Yield 58%, mp 269–270°C. ^1^H NMR: δ 2.69 (s, 3H, CH_3_), 4.45–4.66 and 4.78–4.94 (2m, 2H, CH_2_N), 5.25–5.37 (m, 1H, CHCl), 7.00 (s, 1H, H-3), 7.14–7.58 (m, 9H Ar), 12.60 (br s, 1H, NH disappears with D_2_O). IR (cm^−1^): 2966 (NH). Anal. (C_20_H_17_N_5_ClF) C, H, N.

#### 10

Yield 55%, mp 245–246°C. ^1^H NMR: δ 2.69 (s, 3H, CH_3_), 4.55–4.72 and 4.80–4.98 (2m, 2H, CH_2_N), 5.27–5.39 (m, 1H, CHCl), 6.99 (s, 1H, H-3), 7.16–7.53 (m, 9H Ar). IR (cm^−1^): 3100 (NH). Anal. (C_20_H_17_N_5_Cl_2_) C, H, N.

#### Method B (5)

To a solution of **3a** (3.67g, 10 mmol) in anhydrous toluene (10 ml) phenylethylamine (4.48 g, 40 mmol) was added and the mixture was stirred at room temperature for 48 h. The organic phase was washed with water (2×10 ml), dried (MgSO_4_), filtered, and concentrated under reduced pressure. The crude oil was crystallized by adding a 1∶1 mixture of diethyl ether/petroleum ether (bp 40–60°C), to afford compound **5** as a white solid, yield 60%, mp 125–126°C. ^1^H NMR: δ 1.45 (d, *J* = 6.2, 3H, CH_3_), 1.50 (d, *J* = 6.2, 3H, CH_3_), 2.97 (t, *J* = 6.8, 2H, CH_2_Ar), 3.83 (q, *J* = 6.8, 2H, CH_2_NH), 3.91–4.08 (m, 1H, CHS), 4.63–4.92 (m, 2H, CH_2_N), 5.45–5.58 (m, 1H, CHCl), 7.13–7.44 (m, 10H Ar), 7.67 (s, 1H, H-3). IR (cm^−1^): 3243 (NH). Anal. (C_24_H_26_N_5_ClS) C, H, N, S.

### Pharmacologic inhibitors, Enzymes and Proteins

**Figure 7:**
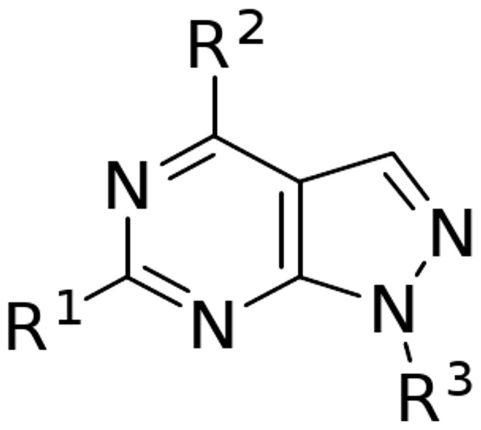
Structure of a pyrazolo-[3,4-d]-pyrimidine.

The dual kinase inhibitors were prepared as a 10 mmol/l stock solution in DMSO. The allocation of the substituents around the pyrazolo[3,4-d]pyrimidine (Fig. 7) is listed in table 1.

Imatinib mesylate and Dasatinib were kindly supplied by Selleck Chemicals LLC (Houston, TX, USA).

Baculovirus-produced recombinant purified his-tagged active against human Src and Abl were purchased from Upstate (Lake Placid, NY, USA).

### Cell lines

Three human and eleven murine cancer cell lines were used. The human NSCLC cell line A549 (ACC 107) and the human colorectal carcinoma cell line CaCo2 (ACC 169) were obtained from Deutsche Sammlung von Mikroorganismen und Zellkulturen (DSMZ, Braunschweig, Germany). The human hepatocellular carcinoma cell line HepG2 (HB-8065) was obtained from American Type Culture Collection (Manassas, VA, USA). The 11 murine NSCLC cell lines were established as described recently [90]. Cells were grown in monolayer cultures in DMEM containing 10% foetal calf serum, 4 mmol/l L-glutamine and 2% Penicillin/Streptomycin at 37°C in a humidified atmosphere of 95% air and 5% CO_2_.

### Enzymatic assays

Src activity was measured in a filter-binding assay using the Src Assay Kit (Upstate, Lake Placid, NY, USA), according to the manufacturer's protocol, using the specific Src peptide substrate [KVEKIGEGTYGVVYK] in the presence of 0.125 pmol of Src and 0.160 pmol of [γ-^32^P]ATP. Unlabelled ATP was added to reach the final concentrations as indicated in the figure legends. Abl activity was measured in a filter-binding assay using an Abl specific peptide substrate (Abtide, Upstate). Reaction conditions were (in a final volume of 10 µl): 25 mM Tris-HCl pH 7.5, 1 mM DTT, 0.012 µM [γ-^32^P]ATP, 0.022 µM c-Abl. Unlabelled ATP/Mg^++^ (1∶1 M/M) mix was added to reach the final ATP concentrations as indicated in the figure legends. Reactions were incubated 10 min at 30°C.The samples (9 µl) were spotted on paper cellulose filters which were washed according to the manufacturer's protocol. Filter-bound radioactivity was measured by liquid scintillation with a Microbeta-Trilux apparatus (PerkinElmer, Waltham, MA, USA).

### Kinetic analysis

Dose-response curves were computer simulated by fitting the data to
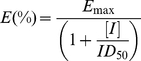
(1)where E(%) is the fraction of the enzyme activity measured in the presence of the inhibitor, E_max_ is the activity in the absence of the inhibitor, [I] is the inhibitor concentration, and ID_50_ is the inhibitor concentration at which E(%) = 0.5E_max_.

The ID_50_ were converted to *K*i according to a competitive mechanism with respect to the substrate ATP. The second substrate of the reaction (the peptide) was kept at saturating concentrations (4-fold higher than its K_m_). Since (i) the ATP concentration was limiting, i.e., [ATP], K_m_(ATP), and (ii) the enzyme concentration was not negligible with respect to the ATP concentration, the classical Cheng-Prusoff relationship was not applicable. Consequently, *K*i values were calculated according to
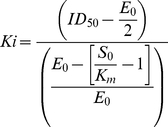
(2)where S_0_ is the concentration of the competing substrate (ATP) and E_0_ is the concentration of the enzyme. Each experiment was done in triplicate and mean values were used for the interpolation. Curve fitting was performed with the program GraphPad Prism.

### MTS-assay

Cell culture viability was monitored by CellTiter96® AQueous One Solution Cell Proliferation Assay (Promega GmbH, Mannheim, Germany). The cells were seeded in triplicates in 96-well microtitre plates and treated with the dual kinase inhibitors or vehicle (0.2% DMSO). After 24 h or 96 h of treatment the MTS tetrazolium reagent was added to the cells and incubated for 1 h. Absorbance at 490 nm was assayed with a Victor3 Wallac 1420 Multilabel counter (PerkinElmer, Waltham, MA, USA). Background absorbance (medium only) was subtracted and the data given as percent of DMSO treated vehicle controls.

### Cell cycle analysis

Cells were seeded in triplicates in 12-well plates and treated at the calculated IC_50_ concentrations of dual kinase inhibitors or vehicle (0.2% DMSO) determined after daily treatment for 96 h. CycleTEST™ PLUS DNA Reagent Kit (Becton Dickinson GmbH, Heidelberg, Germany) for cell cycle analysis was used, according to the instructions of the manufacturer. Briefly, the cells were harvested with trypsin, washed with PBS, and resuspended in CycleTEST Buffer. The cells were centrifuged and resuspended in solution A. After incubation for 10 min at room temperature, solution B was added and incubated. After adding solution C, the samples were incubated at 4°C protected from light. The samples were stored on ice and protected from light until analysis by FACScan flow cytometer (Becton Dickinson GmbH, Heidelberg, Germany).

### Caspase activity assay

The cells were seeded in triplicates in 96-well white-walled microtitre plates and treated with the IC_50_ concentrations of dual kinase inhibitors and vehicle for 24 h or 96 h. According to instructions, Caspase-Glo® 3/7 assay (Promega GmbH, Mannheim, Germany) was used for monitoring caspase activity. Briefly, the same volume Caspase-Glo reagent as culture medium was added and incubated 1 h at room temperature. Luminescence was read with a Victor3 Wallac 1420 Multilabel counter (PerkinElmer, Rodgau, Germany). Background absorbance (medium only) was subtracted and the data given as percent of vehicle controls.

### Western blot

Western blot analysis was carried out after treatment of cell cultures at IC_50_ concentrations for 96 h and compared to untreated cells. For the Western Blot analysis, cells were rinsed and lysed as previously described [91]. An amount of 25 µg protein was used for SDS-PAGE. Antibodies for c-Abl (sc-131), Actin (sc-1616), Cdc2-p34 (sc-8395), EPS15 (sc-1840), FAK (sc-558), c-Fos (sc-52), p38 (sc-7972), p53 (sc-6243) and STAT5 (sc-835x) were obtained from Santa Cruz (Santa Cruz Biotechnology, Heidelberg, Germany), while c-Src (2109) and phospho-Src Y416 (2101) were purchased from Cell Signaling (Cell Signaling Technology, Frankfurt a. M., Germany). The EGFR (06-847), phospho-EGFR Y992 (07-821) and phospho-EGFR Y1045 (04-284) stem from Upstate (Upstate, Schwalbach, Germany) and the hnRNP-K (A300-676A) from Bethyl Labs (Bethyl Laboratories, Montgomery, Texas, USA). Finally p73 (IMG-246) was supplied by Imgenex (Imgenex, Hamburg, Germany). Depending on the primary antibody the secondary antibodies were either horseradish peroxidase-conjugated anti-mouse (AP 160P), anti-rabbit (AP 132P) or anti-sheep (AP 147P) (Chemicon, Schwalbach, Germany).

Semi-quantitative analysis were carried out with Kodak 1D Image Analysis Software in reference to the vehicle treated control cells (see supplementary table S7).

### Whole genome expression analysis

Cells were cultured in 25 cm^2^ culture flasks and treated at IC_50_ concentrations of the dual kinase inhibitors for 96 h. The cells were harvested and RNA was isolated using RNeasy Mini Kit (Qiagen, Hilden, Germany) according to the instructions of the manufacturer. The isolated RNA (250 ng) was processed further using standard protocols as described recently [92] with the exception of the 3′ IVT Express Kit (Affymetrix, High Wycombe, UK) that was utilized according to instructions. Quality control of cRNA was done with the Agilent 2100 Bioanalyzer (Agilent Tech according to manufacturer's protocols. Following fragmentation, cRNA of each sample was hybridized to Affymetrix GeneChip® Mouse Genome 430 2.0 or Human Genome U133 Plus 2.0 and scanned with an Affymetrix 3000 7G scanner.

The resultant data was normalized by Probe Logarithmic Intensity Error Estimation (PLIER) algorithm. Statistical analysis was carried out with the ArrayTrack software (National Center for Toxicological Research/FDA, Jefferson, AR, USA) using SAM algorithm.

### Stem cell marker expression

The gene expression of the stem cell markers *Cd34*, *Cd24a*, *Cd44*, *Cd133*, *Thy1/Cd90*, *Pdpn*, *Nes* and *Dlg7* was determined as a ratio of transcript expression when compared to healthy respiratory epithelium of non-transgenic murine alveolar type II cells. For human cancer cell lines HepG2, CaCo2 and A549 the expression was determined relatively to healthy human hepatocytes, colorectal cells and bronchial epithelial cells, respectively.

## Supporting Information

Figure S1 Network of cell line A549 around c-Abl, EGFR, c-Met and c-Src after treatment with compound Si162. Displayed are molecules that were adjusted after treatment and described in context with the tyrosine kinases c-Abl, EGFR, c-Met and c-Src. In detail the symbols mean: red genes (repressed), green genes (induced), white genes (not adjusted), arrows (direct interactions), broken arrows (indirect interactions).
(0.06 MB PDF)

Figure S2 Network of cell line A2C12 around c-Abl, EGFR, c-Met and c-Src after treatment with compound Si162. Displayed are molecules that were adjusted after treatment and described in context with the tyrosine kinases c-Abl, EGFR, c-Met and c-Src. In detail the symbols mean: red genes (repressed), green genes (induced), white genes (not adjusted), arrows (direct interactions), broken arrows (indirect interactions).
(0.05 MB PDF)

Figure S3 Network of cell line GammaA3 around c-Abl, EGFR, c-Met and c-Src after treatment with compound Si162. Displayed are molecules that were adjusted after treatment and described in context with the tyrosine kinases A: c-Abl, B: EGFR, C: c-Met and D: c-Src. In detail the symbols mean: red genes (repressed), green genes (induced), white genes (not adjusted), arrows (direct interactions), broken arrows (indirect interactions).
(0.20 MB PDF)

Figure S4 Network of cell line CaCo2 around c-Abl, EGFR, c-Met and c-Src after treatment with compound Si162. Displayed are molecules that were adjusted after treatment and described in context with the tyrosine kinases c-Abl, EGFR, c-Met and c-Src. In detail the symbols mean: red genes (repressed), green genes (induced), white genes (not adjusted), arrows (direct interactions), broken arrows (indirect interactions).
(0.03 MB PDF)

Figure S5 Network of cell line HepG2 around c-Abl, EGFR, c-Met and c-Src after treatment with compound Si162. Displayed are molecules that were adjusted after treatment and described in context with the tyrosine kinases A: c-Abl and c-Src as well as B: EGFR and c-Met. In detail the symbols mean: red genes (repressed), green genes (induced), white genes (not adjusted), arrows (direct interactions), broken arrows (indirect interactions).
(0.13 MB PDF)

Figure S6 Network of cell line A549 around c-Abl, EGFR, c-Met and c-Src after treatment with compound Si135. Displayed are molecules that were adjusted after treatment and described in context with the tyrosine kinases c-Abl, EGFR, c-Met and c-Src. In detail the symbols mean: red genes (repressed), green genes (induced), white genes (not adjusted), arrows (direct interactions), broken arrows (indirect interactions).
(0.02 MB PDF)

Figure S7 Network of cell line A2C12 around c-Abl, EGFR, c-Met and c-Src after treatment with compound Si135. Displayed are molecules that were adjusted after treatment and described in context with the tyrosine kinases c-Abl, EGFR, c-Met and c-Src. In detail the symbols mean: red genes (repressed), green genes (induced), white genes (not adjusted), arrows (direct interactions), broken arrows (indirect interactions).
(0.02 MB PDF)

Figure S8 Network of cell line GammaA3 around c-Abl, EGFR, c-Met and c-Src after treatment with compound Si135. Displayed are molecules that were adjusted after treatment and described in context with the tyrosine kinases c-Abl, EGFR, c-Met and c-Src. In detail the symbols mean: red genes (repressed), green genes (induced), white genes (not adjusted), arrows (direct interactions), broken arrows (indirect interactions).
(0.03 MB PDF)

Figure S9 Network of cell line CaCo2 around c-Abl, EGFR, c-Met and c-Src after treatment with compound Si135. Displayed are molecules that were adjusted after treatment and described in context with the tyrosine kinases c-Abl, EGFR, c-Met and c-Src. In detail the symbols mean: red genes (repressed), green genes (induced), white genes (not adjusted), arrows (direct interactions), broken arrows (indirect interactions).
(0.04 MB PDF)

Figure S10 Network of cell line HepG2 around c-Abl, EGFR, c-Met and c-Src after treatment with compound Si135. Displayed are molecules that were adjusted after treatment and described in context with the tyrosine kinases c-Abl, EGFR, c-Met and c-Src. In detail the symbols mean: red genes (repressed), green genes (induced), white genes (not adjusted), arrows (direct interactions), broken arrows (indirect interactions).
(0.03 MB PDF)

Table S1 Cancer stem cell markers expressed in tested tumour cell lines. Mean values in percent compared to non-transgenic and appropriate controls.
(0.01 MB XLS)

Table S2 Calculated IC50 values after single treatment for 24 h. Values are displayed in µmol/l. n.a.: not applicable. IC50>100 µmol/l.
(0.02 MB XLS)

Table S3 Calculated IC50 values after daily treatment for 96 h. Values are displayed in µM. n.a.: not applicable, as IC50>20 µM. except HepG2 where IC50>100 µM.
(0.02 MB XLS)

Table S4 Cell cycle regulation. The cells were treated with their IC50 concentrations for 96 h. All values in %. N.a.: not applicable.
(0.03 MB XLS)

Table S5 Selected significantly regulated genes after treatment with dual kinase inhibitor Si135. Significantly regulated genes were analyzed with SAM. All values are displayed as log2 of fold change.
(0.02 MB XLS)

Table S6 Selected significantly regulated genes after treatment with dual kinase inhibitor Si162. Significantly regulated genes were analyzed with SAM. All values are displayed as log2 of fold change.
(0.03 MB XLS)

Table S7 Semi-quantitative analysis of protein expression. Semi-quantitative analysis of protein expression in reference of untreated control cells. Band intensities obtained by Western blot analysis were computed by Kodak 1D Image Analysis software. All values are displayed as percent of the vehicle treated control cells. N.A. not applicable.
(0.02 MB XLS)
