# Supplementary figures and images for: Molecular Characterization of c-Abl/c-Src Kinase Inhibitors Targeted against Murine Tumour Progenitor Cells that Express Stem Cell Markers
Source: PLoS One. 2010 Nov 30;5(11):e14143. doi: 10.1371/journal.pone.0014143 (PMC2994747; doi:10.1371/journal.pone.0014143)

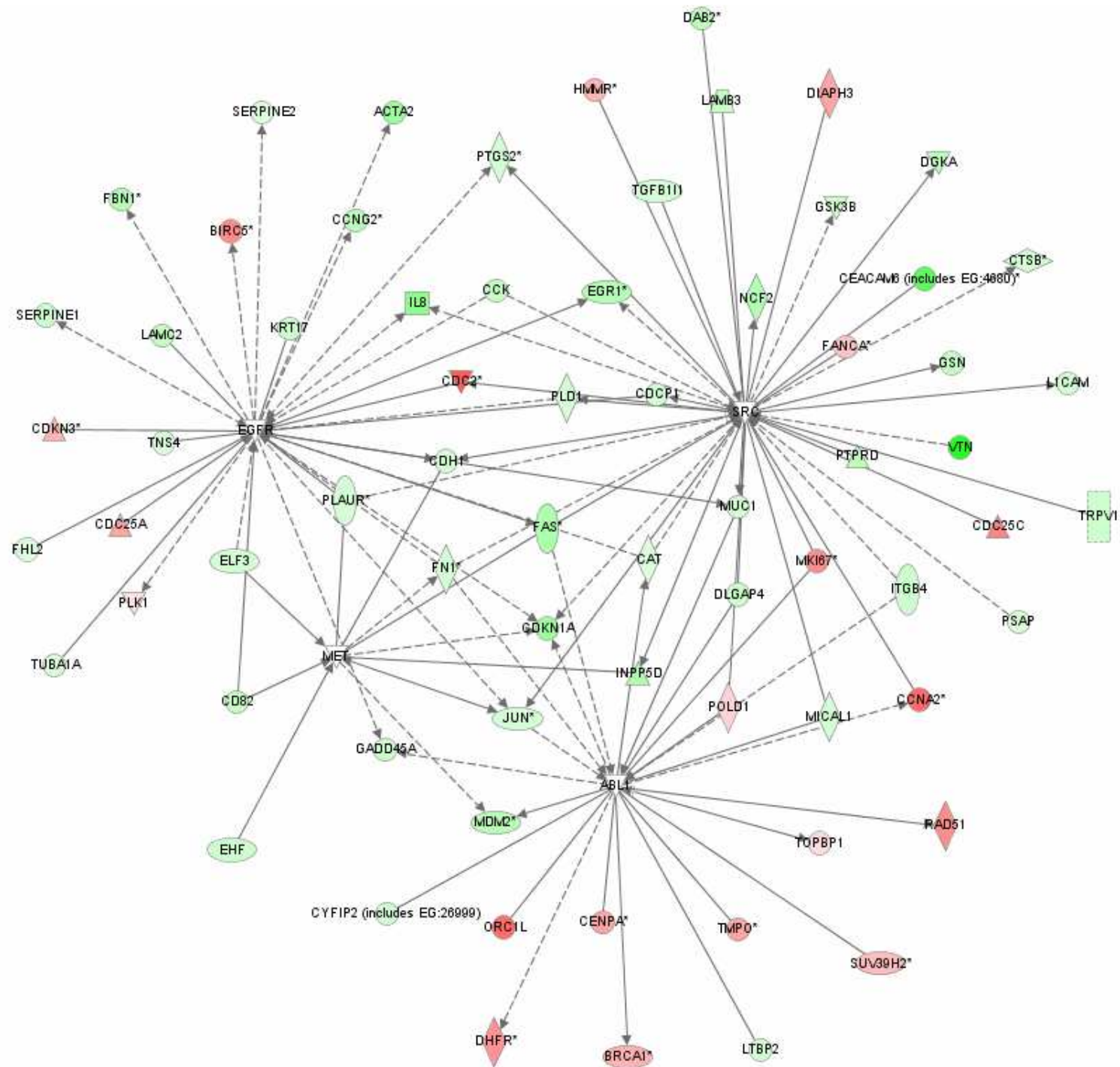

Supplement: Figure S1 — Network of cell line A549 around c-Abl, EGFR, c-Met and c-Src after treatment with compound Si162. Displayed are molecules that were adjusted after treatment and described in context with the tyrosine kinases c-Abl, EGFR, c-Met and c-Src. In detail the symbols mean: red genes (repressed), green genes (induced), white genes (not adjusted), arrows (direct interactions), broken arrows (indirect interactions). (0.06 MB PDF) [file pone.0014143.s001.pdf]

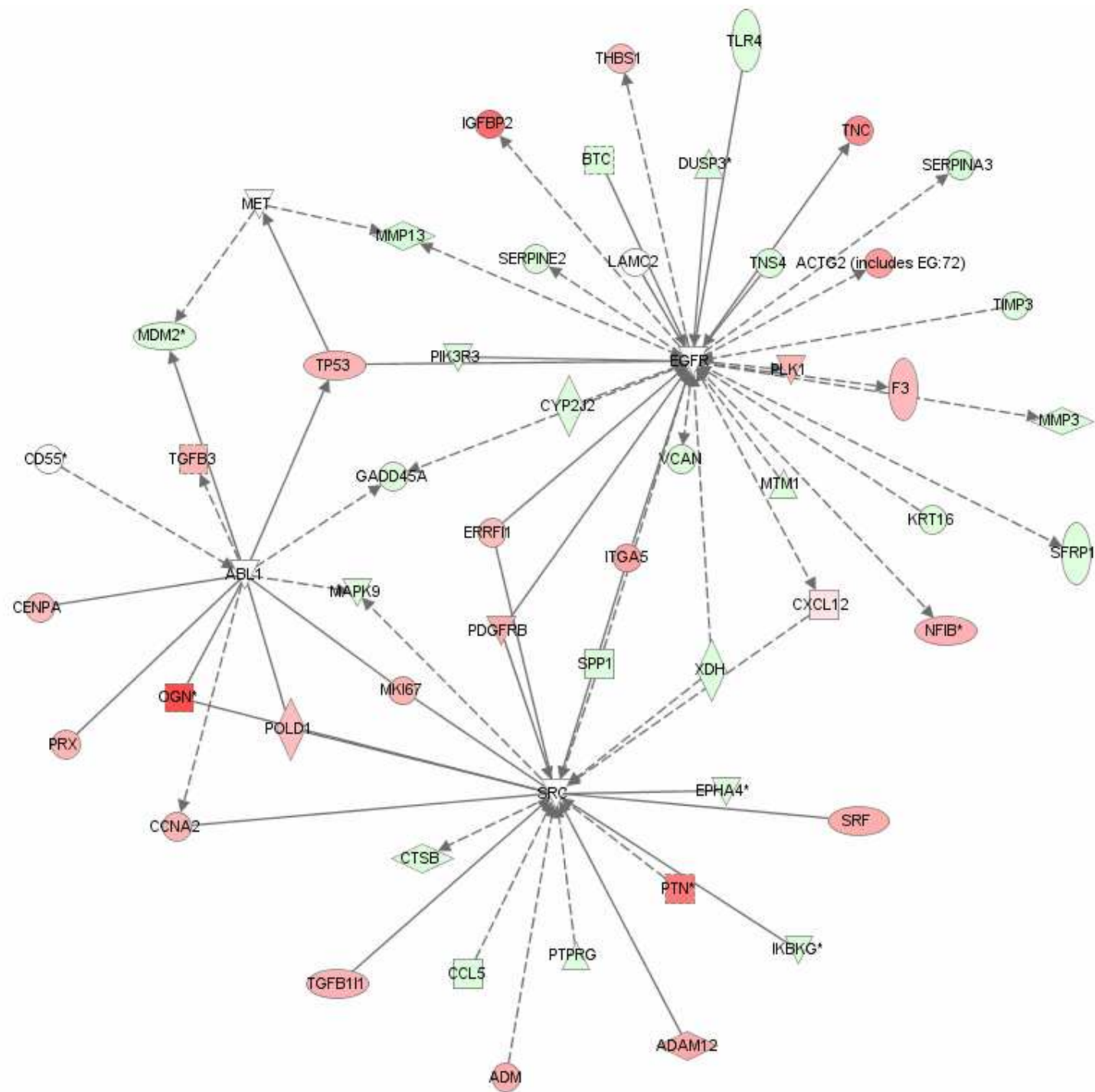

Supplement: Figure S2 — Network of cell line A2C12 around c-Abl, EGFR, c-Met and c-Src after treatment with compound Si162. Displayed are molecules that were adjusted after treatment and described in context with the tyrosine kinases c-Abl, EGFR, c-Met and c-Src. In detail the symbols mean: red genes (repressed), green genes (induced), white genes (not adjusted), arrows (direct interactions), broken arrows (indirect interactions). (0.05 MB PDF) [file pone.0014143.s002.pdf]

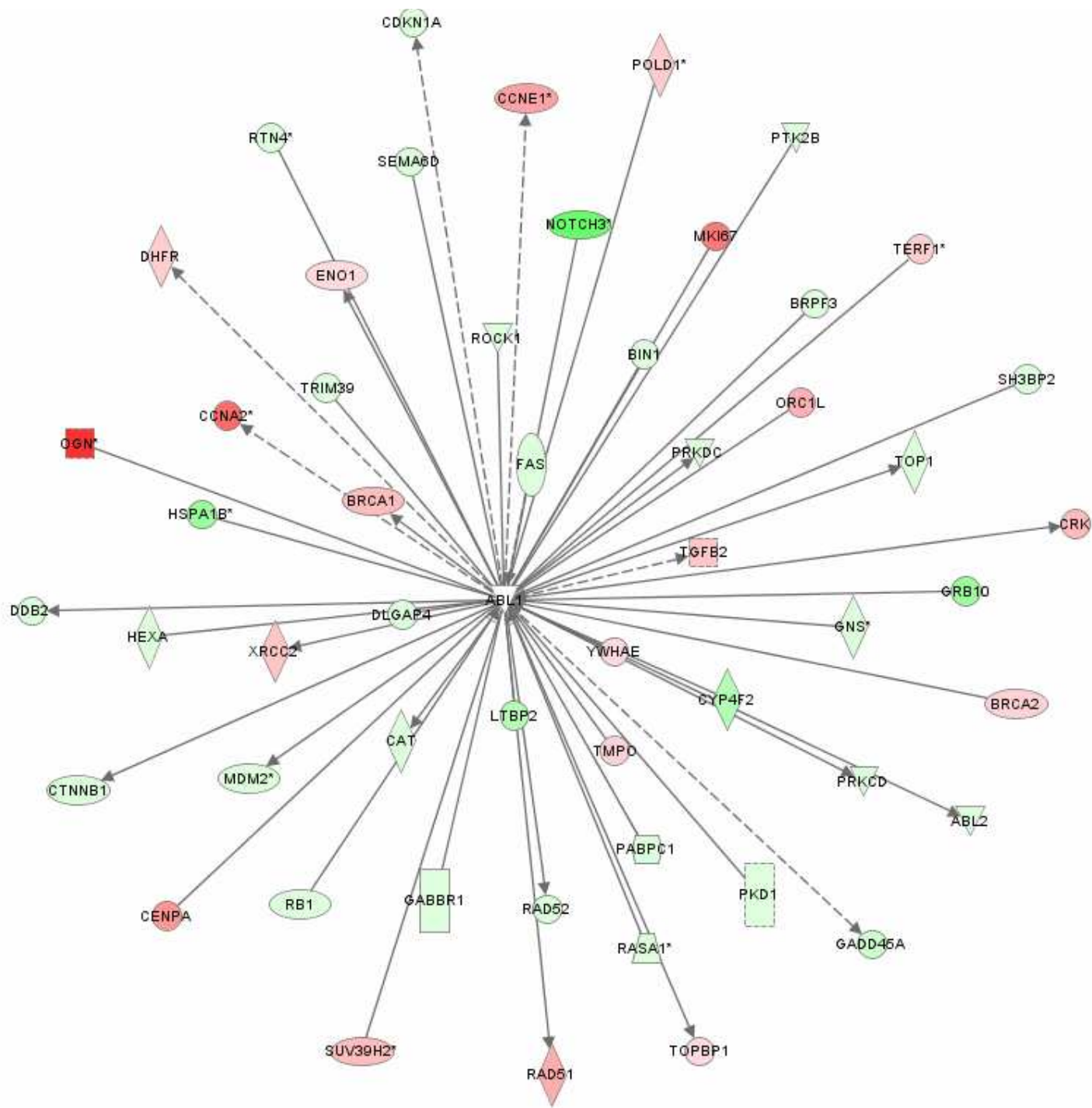

A

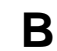

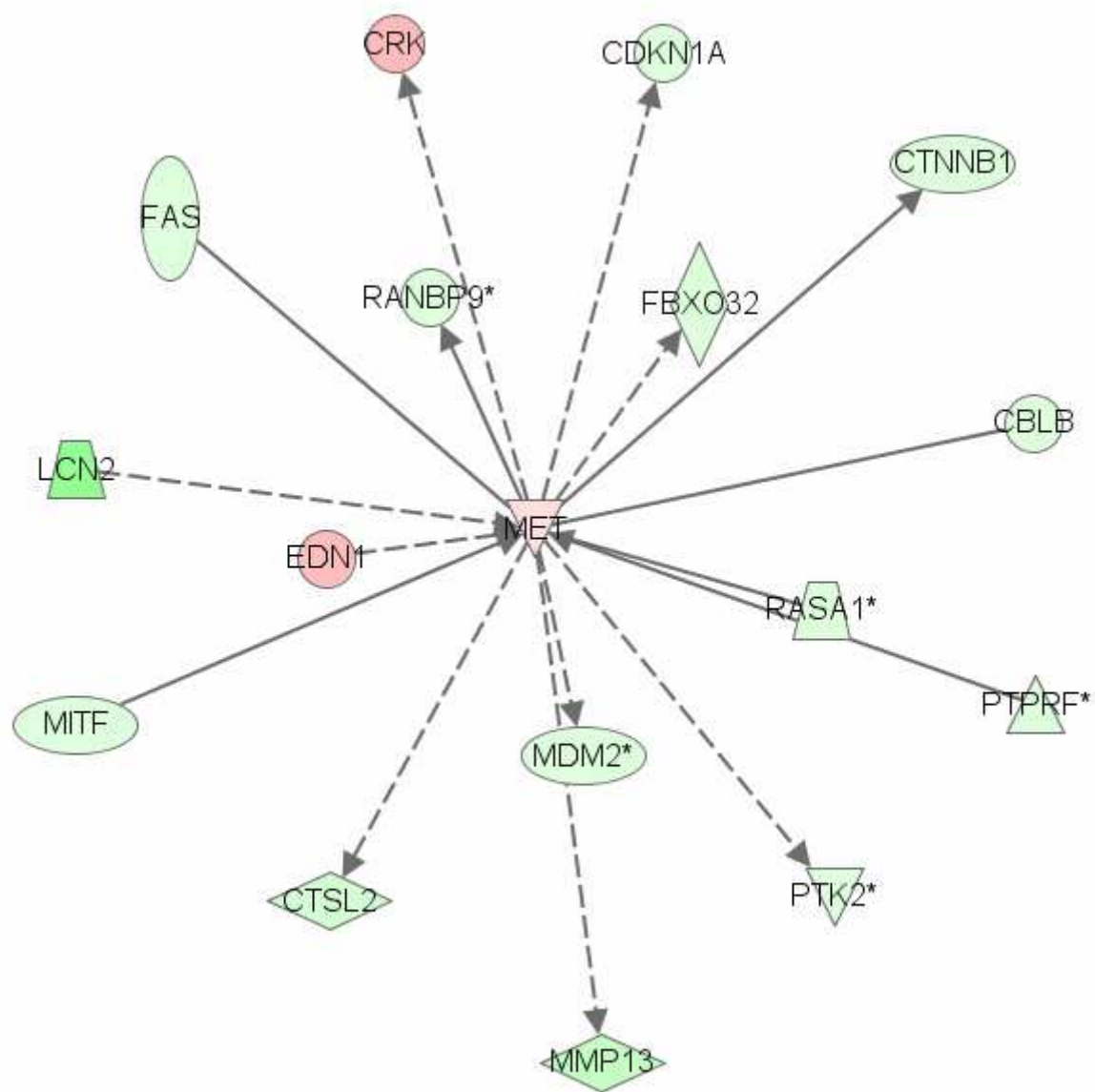

**C**

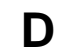

Supplement: Figure S3 — Network of cell line GammaA3 around c-Abl, EGFR, c-Met and c-Src after treatment with compound Si162. Displayed are molecules that were adjusted after treatment and described in context with the tyrosine kinases A: c-Abl, B: EGFR, C: c-Met and D: c-Src. In detail the symbols mean: red genes (repressed), green genes (induced), white genes (not adjusted), arrows (direct interactions), broken arrows (indirect interactions). (0.20 MB PDF) [file pone.0014143.s003.pdf]

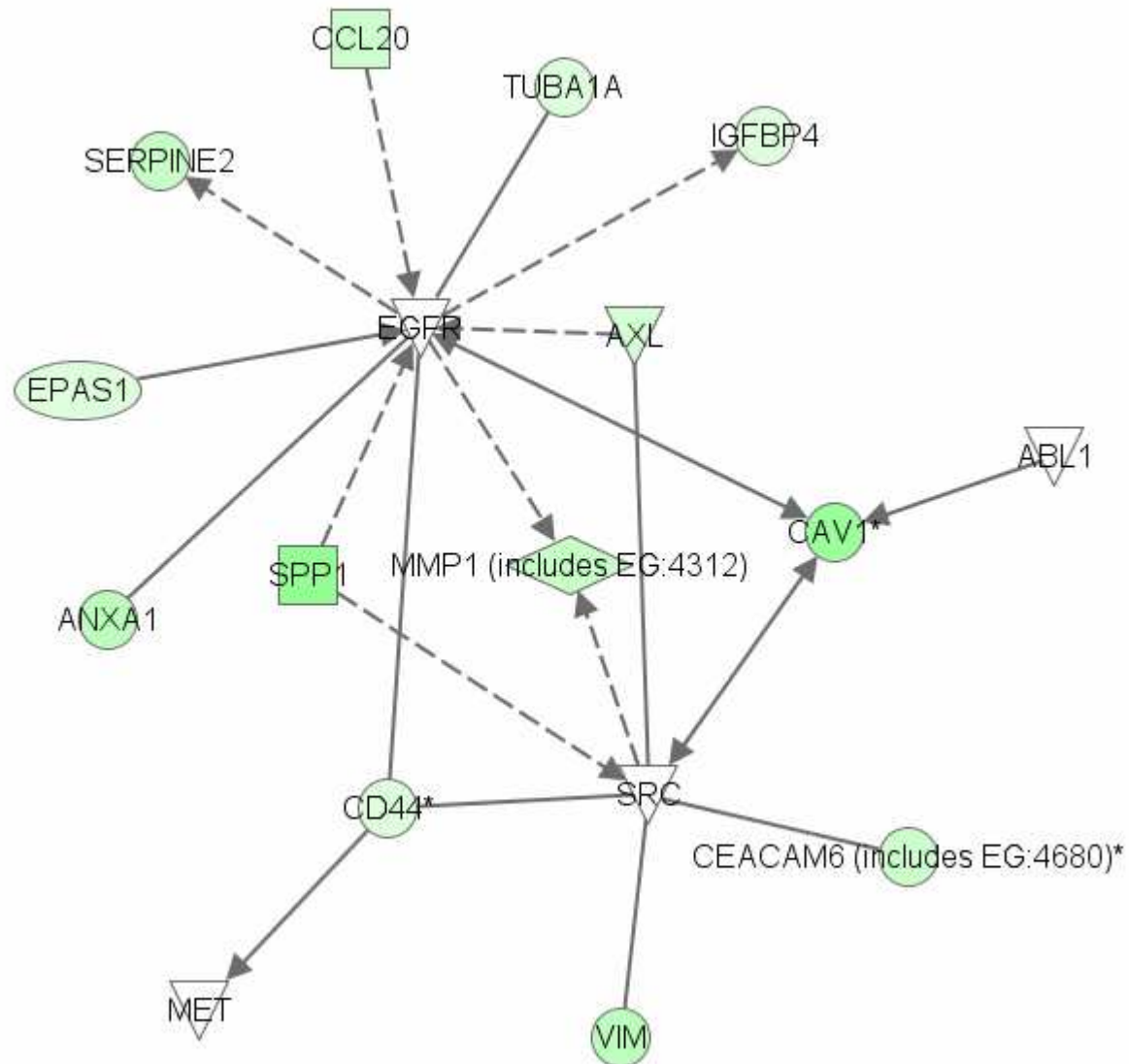

Supplement: Figure S4 — Network of cell line CaCo2 around c-Abl, EGFR, c-Met and c-Src after treatment with compound Si162. Displayed are molecules that were adjusted after treatment and described in context with the tyrosine kinases c-Abl, EGFR, c-Met and c-Src. In detail the symbols mean: red genes (repressed), green genes (induced), white genes (not adjusted), arrows (direct interactions), broken arrows (indirect interactions). (0.03 MB PDF) [file pone.0014143.s004.pdf]

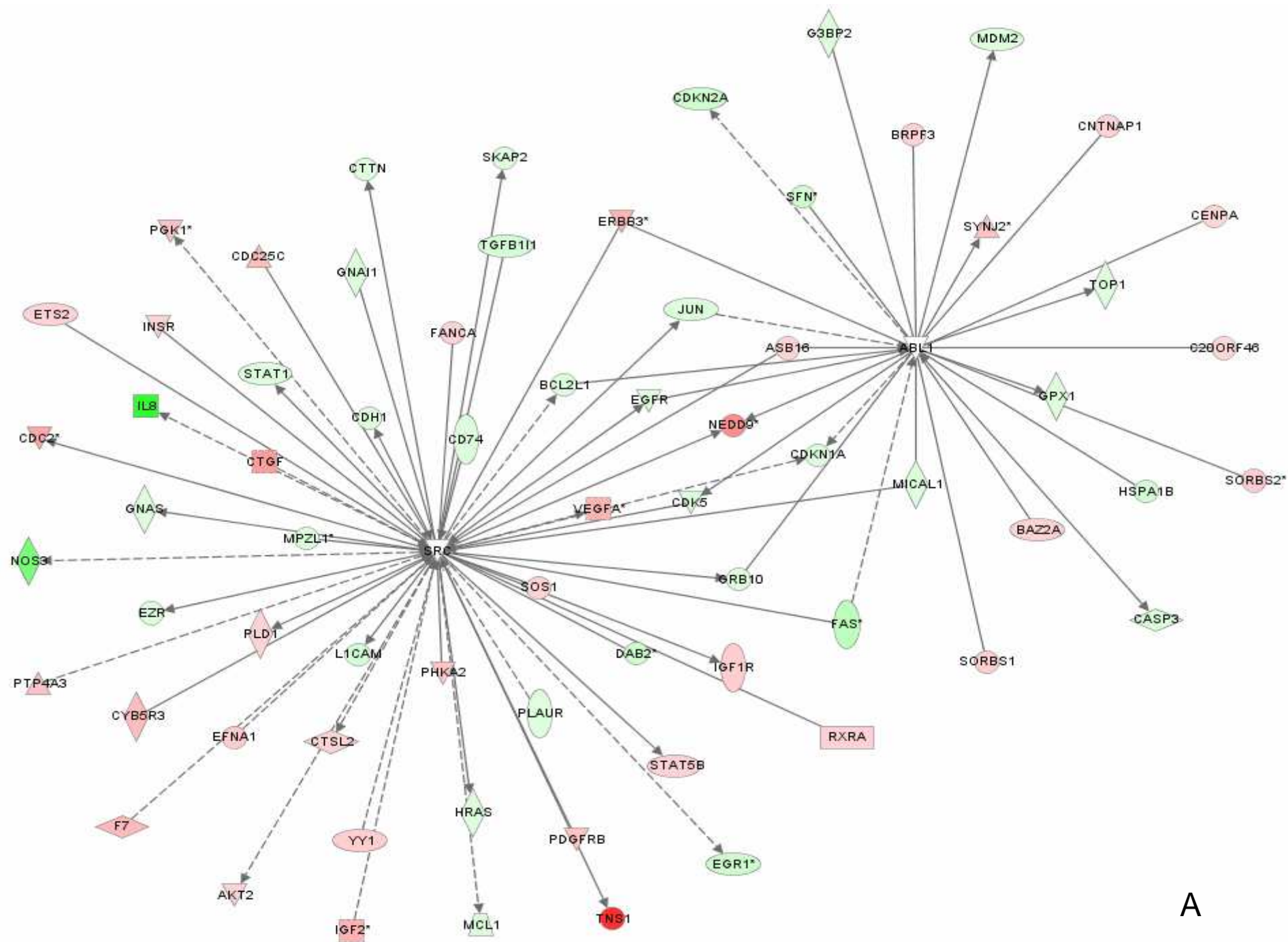

A

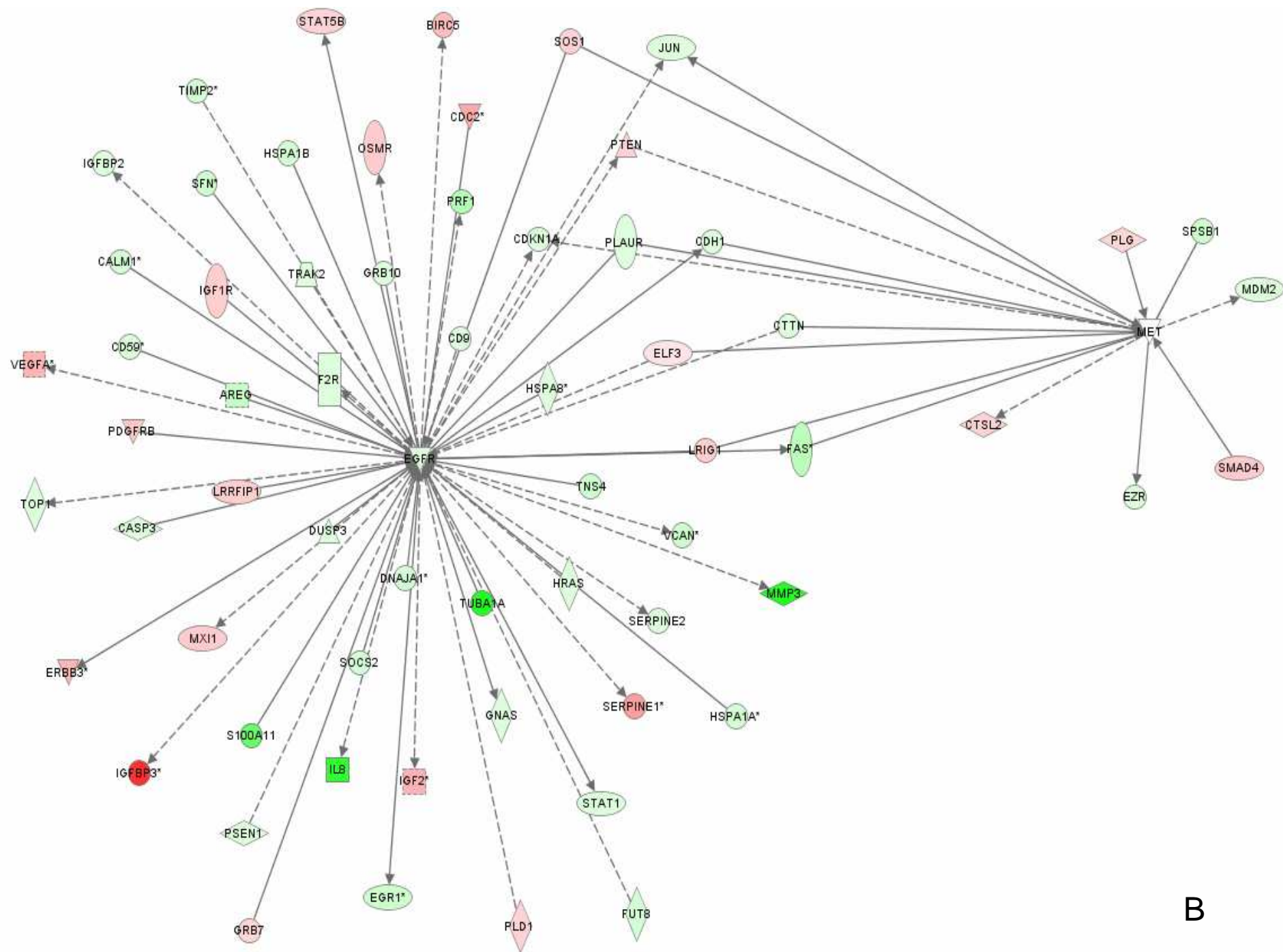

B

Supplement: Figure S5 — Network of cell line HepG2 around c-Abl, EGFR, c-Met and c-Src after treatment with compound Si162. Displayed are molecules that were adjusted after treatment and described in context with the tyrosine kinases A: c-Abl and c-Src as well as B: EGFR and c-Met. In detail the symbols mean: red genes (repressed), green genes (induced), white genes (not adjusted), arrows (direct interactions), broken arrows (indirect interactions). (0.13 MB PDF) [file pone.0014143.s005.pdf]

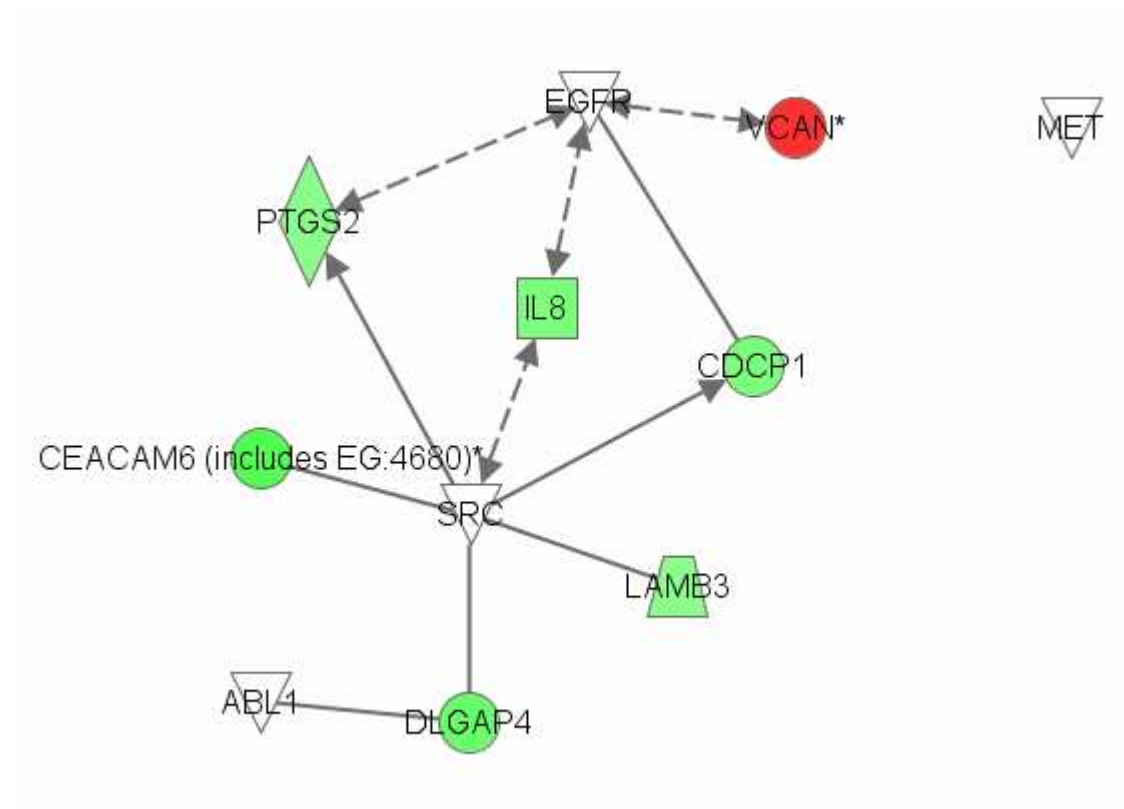

Supplement: Figure S6 — Network of cell line A549 around c-Abl, EGFR, c-Met and c-Src after treatment with compound Si135. Displayed are molecules that were adjusted after treatment and described in context with the tyrosine kinases c-Abl, EGFR, c-Met and c-Src. In detail the symbols mean: red genes (repressed), green genes (induced), white genes (not adjusted), arrows (direct interactions), broken arrows (indirect interactions). (0.02 MB PDF) [file pone.0014143.s006.pdf]

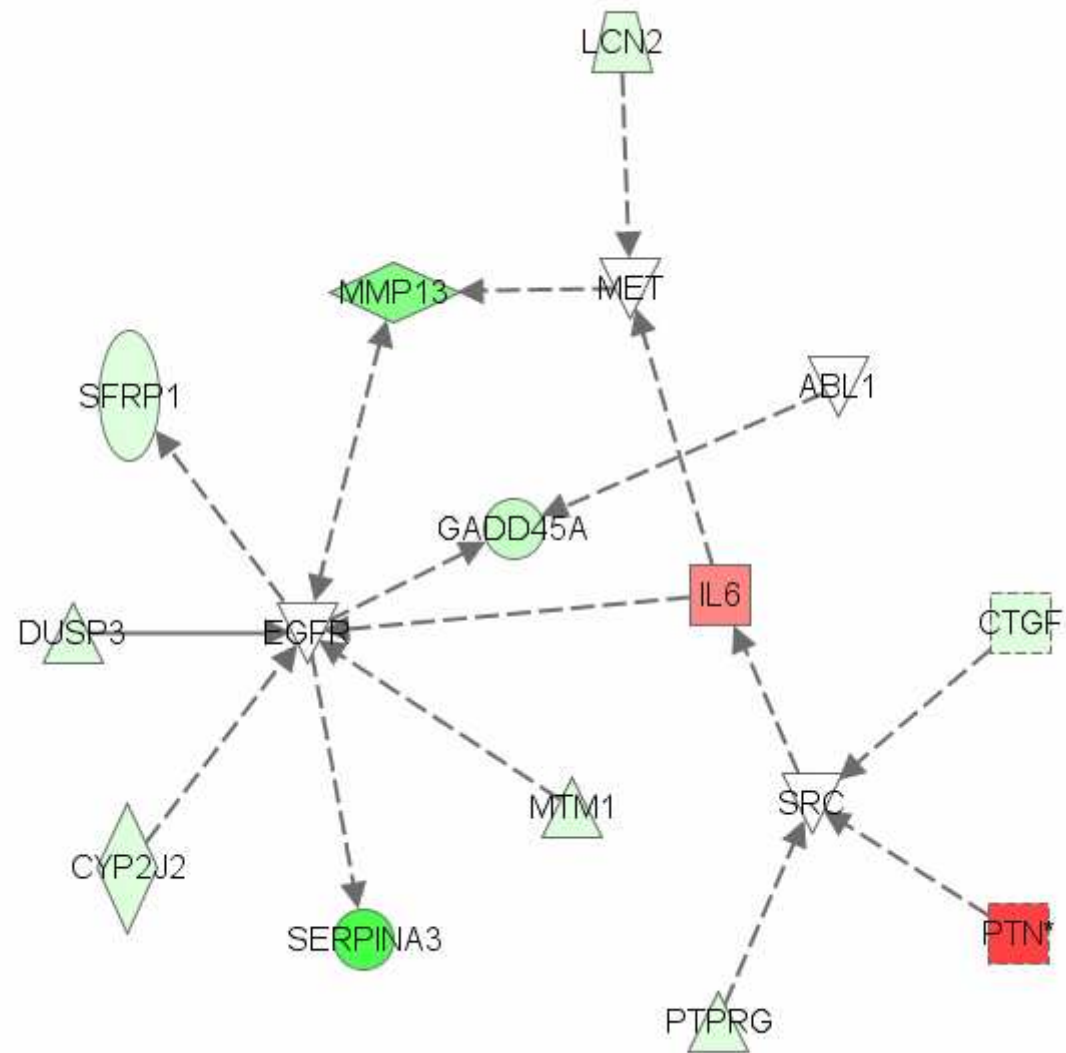

Supplement: Figure S7 — Network of cell line A2C12 around c-Abl, EGFR, c-Met and c-Src after treatment with compound Si135. Displayed are molecules that were adjusted after treatment and described in context with the tyrosine kinases c-Abl, EGFR, c-Met and c-Src. In detail the symbols mean: red genes (repressed), green genes (induced), white genes (not adjusted), arrows (direct interactions), broken arrows (indirect interactions). (0.02 MB PDF) [file pone.0014143.s007.pdf]

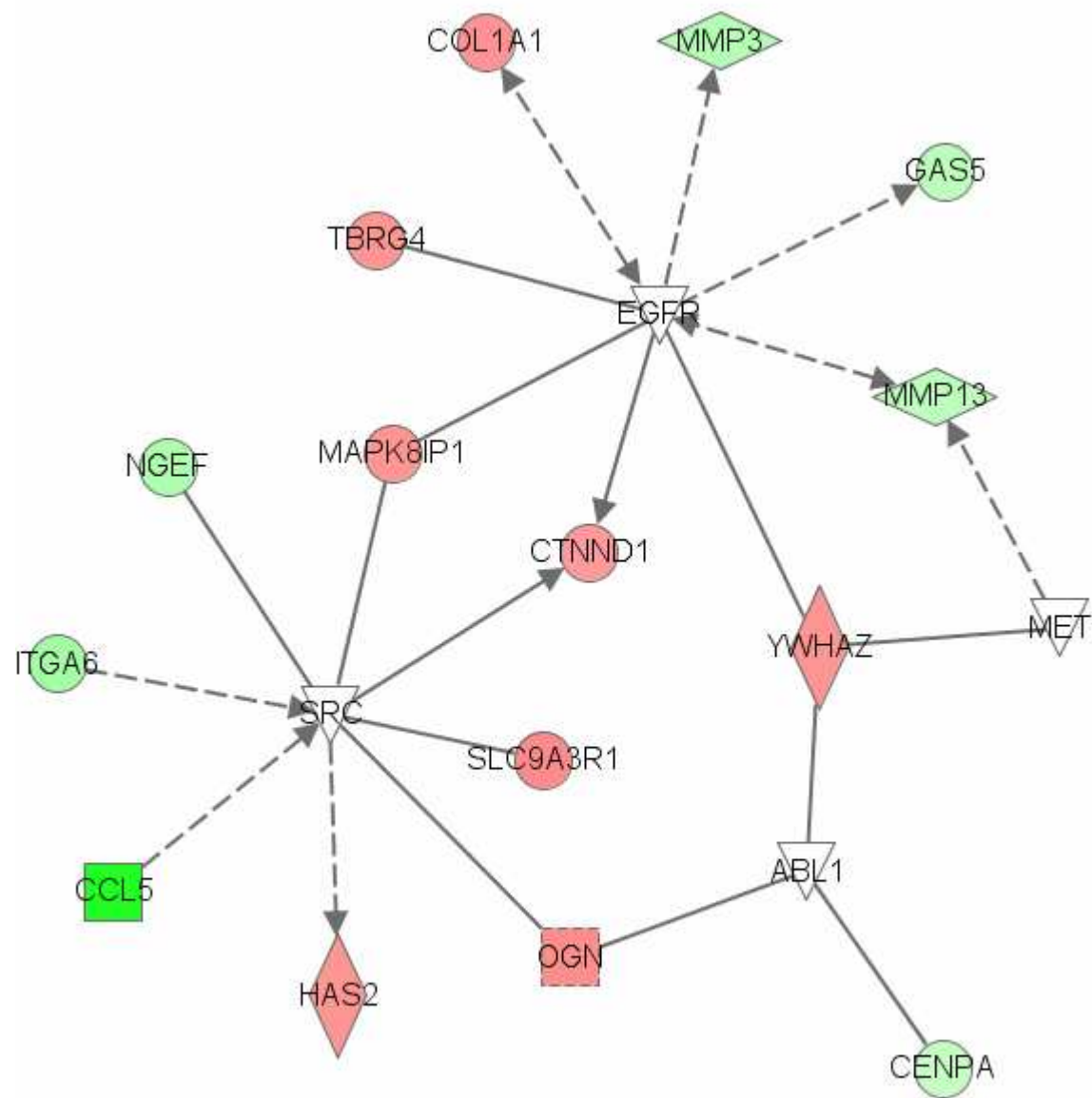

Supplement: Figure S8 — Network of cell line GammaA3 around c-Abl, EGFR, c-Met and c-Src after treatment with compound Si135. Displayed are molecules that were adjusted after treatment and described in context with the tyrosine kinases c-Abl, EGFR, c-Met and c-Src. In detail the symbols mean: red genes (repressed), green genes (induced), white genes (not adjusted), arrows (direct interactions), broken arrows (indirect interactions). (0.03 MB PDF) [file pone.0014143.s008.pdf]

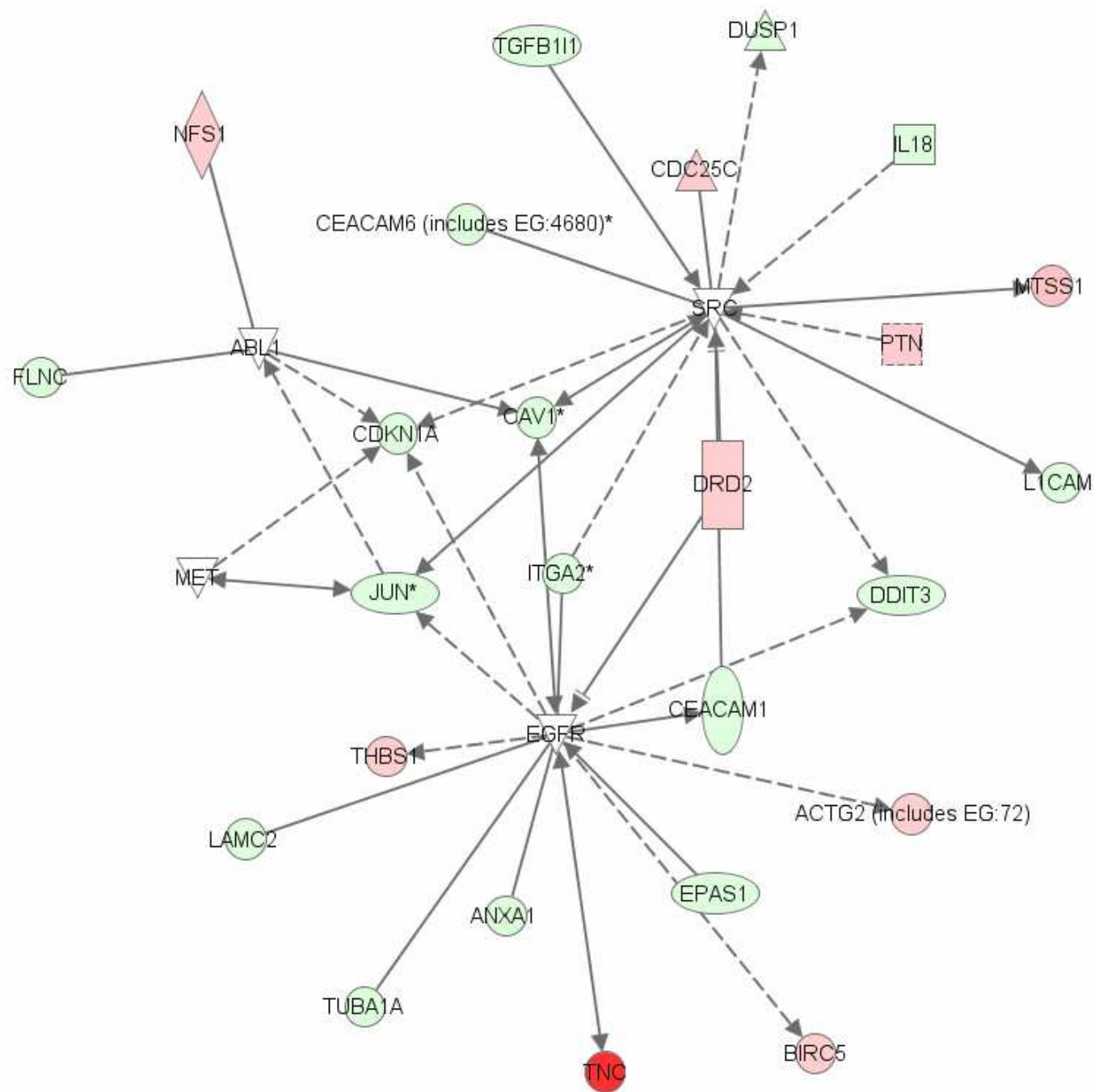

Supplement: Figure S9 — Network of cell line CaCo2 around c-Abl, EGFR, c-Met and c-Src after treatment with compound Si135. Displayed are molecules that were adjusted after treatment and described in context with the tyrosine kinases c-Abl, EGFR, c-Met and c-Src. In detail the symbols mean: red genes (repressed), green genes (induced), white genes (not adjusted), arrows (direct interactions), broken arrows (indirect interactions). (0.04 MB PDF) [file pone.0014143.s009.pdf]

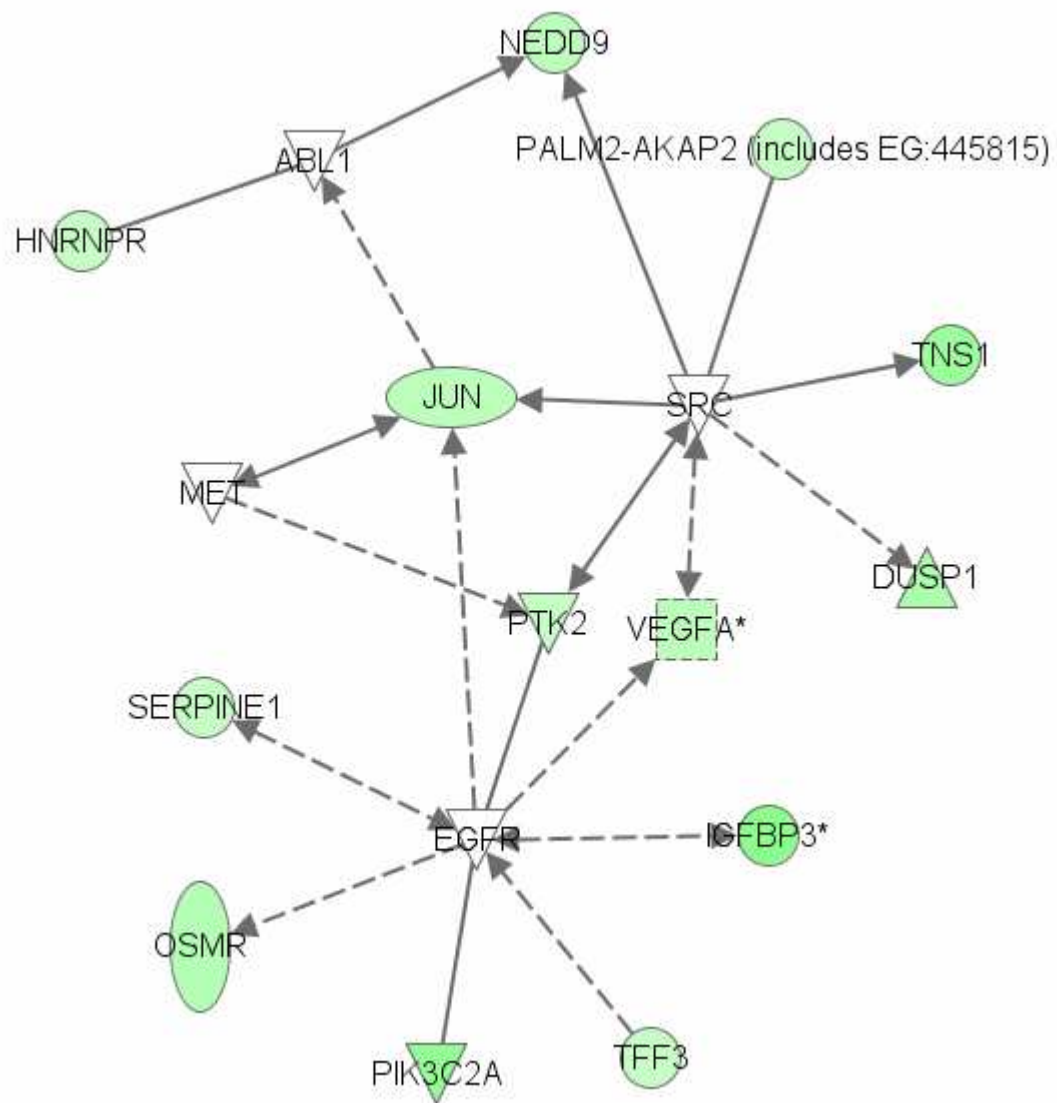

Supplement: Figure S10 — Network of cell line HepG2 around c-Abl, EGFR, c-Met and c-Src after treatment with compound Si135. Displayed are molecules that were adjusted after treatment and described in context with the tyrosine kinases c-Abl, EGFR, c-Met and c-Src. In detail the symbols mean: red genes (repressed), green genes (induced), white genes (not adjusted), arrows (direct interactions), broken arrows (indirect interactions). (0.03 MB PDF) [file pone.0014143.s010.pdf]
